# The EXTREME Regimen Associating Cetuximab and Cisplatin Favors Head and Neck Cancer Cell Death and Immunogenicity with the Induction of an Anti-Cancer Immune Response

**DOI:** 10.3390/cells11182866

**Published:** 2022-09-14

**Authors:** Justine De Azevedo, Jana Mourtada, Cyril Bour, Véronique Devignot, Philippe Schultz, Christian Borel, Erwan Pencreach, Georg Mellitzer, Christian Gaiddon, Alain C. Jung

**Affiliations:** 1 Laboratory Streinth, Université de Strasbourg-Inserm, UMR_S 1113 IRFAC, 67200 Strasbourg, France; 2 Laboratoire de Biologie Tumorale, Institut de Cancérologie Strasbourg Europe, 67200 Strasbourg, France; 3 Department of Otorhinolaryngology and Head and Neck Surgery, Hôpitaux Universitaires de Strasbourg, 67200 Strasbourg, France; 4 Department of Medical Oncology, Institut de Cancérologie Strasbourg Europe, 67200 Strasbourg, France; 5 Laboratoire de Biochimie et Biologie Moléculaire, Hôpitaux Universitaires de Strasbourg, 67200 Strasbourg, France

**Keywords:** head and neck squamous cell carcinoma, cetuximab, cisplatin, apoptosis, immunogenic cell death

## Abstract

(1) Background: The first line of treatment for recurrent/metastatic Head and Neck Squamous Cell Carcinoma (HNSCC) has recently evolved with the approval of immunotherapies that target the anti-PD-1 immune checkpoint. However, only about 20% of the patients display a long-lasting objective tumor response. The modulation of cancer cell immunogenicity via a treatment-induced immunogenic cell death is proposed to potentially be able to improve the rate of patients who respond to immune checkpoint blocking immunotherapies. (2) Methods: Using human HNSCC cell line models and a mouse oral cancer syngeneic model, we have analyzed the ability of the EXTREME regimen (combination therapy using the anti-EGFR cetuximab antibody and platinum-based chemotherapy) to modify the immunogenicity of HNSCC cells. (3) Results: We showed that the combination of cetuximab and cisplatin reduces cell growth through both cell cycle inhibition and the induction of apoptotic cell death independently of p53. In addition, different components of the EXTREME regimen were found to induce, to a variable extent, and in a cell-dependent manner, the emission of mediators of immunogenic cell death, including calreticulin, HMGB1, and type I Interferon-responsive chemokines. Interestingly, cetuximab alone or combined with the IC_50_ dose of cisplatin can induce an antitumor immune response in vivo, but not when combined with a high dose of cisplatin. (4) Conclusions: Our observations suggest that the EXTREME protocol or cetuximab alone are capable, under conditions of moderate apoptosis induction, of eliciting the mobilization of the immune system and an anti-tumor immune response in HNSCC.

## 1. Introduction

Head and neck cancer squamous cell carcinoma (HNSCC) are cancers that arise from the mucosal epithelium of the oral cavity, larynx, and pharynx [1]. The principal risk factors for HNSCC are alcohol and tobacco consumption on the one hand, and Human Papillomavirus (HPV) infection on the other hand. They are the sixth most frequent malignancies with ~700,000 new cases being diagnosed worldwide each year [2]. Due to the fact that most tumors are diagnosed at locally-advanced stages [1], as well as to treatment failure despite recent medical progressions [3], the five-year overall survival of patients with HNSCC is poor (<40–50%) [4]. The management of the majority of patients with HNSCC relies on a multimodal approach that involves surgery (in amenable patients), followed by adjuvant radiotherapy or platinum-based (e.g., cisplatin or carboplatin, and 5-fluorouracil) chemoradiotherapy [1]. Cetuximab was FDA-approved in 2006 as a targeted therapy for the management of locally advanced recurrent/metastatic (R/M) HNSCC. The rationale of this therapy relies on the overexpression of the Epidermal Growth Factor Receptor (EGFR) in >90% of HNSCC tumors. The EXTREME phase III clinical trial evaluated the efficacy of the combination of cetuximab and platinum-based chemotherapy (using cisplatin or carboplatin,) as a first-line treatment in patients with R/M HNSCC. This clinical trial showed that the combination in the EXTREME protocol of cetuximab with platinum-based chemotherapy improves both disease-free and overall survival [5,6,7]. Based on this positive outcome, the EXTREME regimen was FDA-approved and became a therapeutic option for the management of patients with R/M HSNCC. The efficiency of the EXTREME protocol could be rationalized by the fact that cells from various molecular subtypes of HNSCC have shown a different degree of response to EGFR blockade [8,9], and that EGFR overexpression has been shown to reduce the cytotoxicity of metal-based drugs [10].

More recently, several immune check-point blocking immunotherapies, which aim to reactivate an anti-tumor immune response, have been approved [11]. Unfortunately, resistance mechanisms to cisplatin and cetuximab are common. They include the overexpression of factors involved in DNA repair or the constitutive, ligand-independent activation of the EGFR pathway, which reduce the benefits of treatments [12]. Furthermore, only a small proportion of patients (<20%) show a tumor response to immune checkpoint-blocking immunotherapies used as monotherapies [13,14]. The immune landscape of the microenvironment (i.e., the nature of immune cells in the microenvironment and their respective proportions) has been proposed to play a role in the tumor response to immune checkpoint inhibiting immunotherapies [15]. Understanding and detecting the variations in the immune cell landscape that can account for a response to immunotherapy is a major goal to improve patient care [16].

The evolution of the cancer immune landscape during tumor progression was previously described by the three Es (Elimination; Equilibrium; Escape) of the cancer immunoediting model [17]. During the “Elimination” phase (when tumor cells are eliminated by the immune cells), tumor-associated antigens (or neoantigens) are up-taken by antigen presenting cells (APCs) like dendritic cells or macrophages phagocyte, which are further cross-presented to cytotoxic CD8+ T lymphocyte (TL) [18,19]. Cytotoxic TLs are the main actors of the anti-cancer immune response: they infiltrate tumors and trigger targeted cell death via the expression perforin and granzymes. Therefore, an “inflamed” or “immuno-suppressive” tumor microenvironment with high infiltration by cytotoxic CD8+ LT is associated with a better patient outcome [20]. Yet, several mechanisms are known to dampen this anti-tumor cytotoxicity and are responsible for the transition from the “Elimination” to the “Equilibrium” and eventually “Escape” phases (during which cancer cells are progressively maintained and escape the immune system). One of these mechanisms relies on the enrichment of the tumor microenvironment with immunosuppressive immune cells (e.g., regulatory T cells (Treg) [21]; pro-tumoral M2 macrophages [22]; myeloid-derived suppressor cells [23]). The microenvironment of HNSCC is known to be frequently “immuno-tolerant” (presence of pro-tumoral M2 macrophages and/or Treg cells) and associated with a poor outcome [11,24]. In addition, cancer cells highjack immune checkpoints to induce cytotoxic LT anergy: for example, the expression of Programmed Death-Ligand 1 (PD-L1) by cancer cells inhibits TLs’ cytotoxic activity upon binding with the Programmed Death-1 (PD-1) receptor and allow immune evasion [17]. Increasing tumor immunogenicity and favorizing an immune-suppressive microenvironment to restore anti-tumor activity is therefore proposed to be an interesting option to improve the efficiency of immunotherapies.

One attractive possibility to achieve this could be to trigger an immunogenic cell death (ICD), which is known to induce an immune response [25]. This particular death cell is characterized by the emission of danger-associated molecular patterns (DAMPs) by dying cells, the activation of APCs upon binding of DAMPs to specific receptors as well as tumor neoantigens uptake, the subsequent activation of a CD8+ TL-based immune response, and the establishment of an immune memory, which eliminates tumor cells [25]. DAMPs are danger signals that are either expressed on the cell surface and act as “eat me” signals for APCs, like the calreticulin (CRT) chaperone protein, or factors that are released in the extracellular space and act as pro-inflammatory chemoattractant signals, like the histone group mobility box (HMGB1) protein [26]. In addition, the secretion of type I interferons also acts as a DAMP and results in the production of the CXCL10 chemokine which is a chemoattractant for cytotoxic TL [27]. It has been shown that several anticancer treatments can induce ICD, such as specific chemotherapies (i.e., oxaliplatin) [28], radiotherapy [28], or even photodynamic therapy [28,29]. In the clinic, inducing ICD in patients could activate an anti-tumor immune response, provoke tumor elimination and provide protection against relapse through an immune memory. Interestingly, cetuximab was shown to induce ICD in colon cancer cells [30].

While cisplatin used alone was previously proposed to modestly induce ICD in HNSCC cell lines [31], the ICD-inducing ability of cetuximab, used either alone or in combination with cisplatin in head and neck cancers, remains to be determined. Therefore, while the protocol EXTREME is used in clinical routine to treat HNSCC patients, its precise impact on the modulation of immunogenicity of HNSCC cells has never been investigated. Based on previous findings showing that cetuximab can elicit ICD in colon cancer [30], we hypothesized that it has similar effects in HNSCC. In addition, we wanted to investigate the precise impact of the EXTREME protocol (i.e., the combination of cetuximab and cisplatin) on cell proliferation and apoptotic cell death, and how this correlates with the induction of ICD. Hence, we first analyzed the biological impact of cetuximab and cisplatin cotreatment on HNSCC cell line models through the analysis of cell cycle and apoptotic cell death. Secondly, we demonstrated the capacity of cetuximab (alone or combined with cisplatin) to induce DAMPs emission. Finally, using prophylactic vaccination of HNSCC syngeneic mouse models, we show that the treatment with cetuximab provides animals with anti-tumor immune protection.

## 2. Materials and Methods

### 2.1. Cell Lines and Reagents

The SQ20B cells originate from a laryngeal tumor, express mutated *TP53*, and are a kind gift from Dr. Pierre Bischoff. The CAL27 cell line originates from a carcinoma of the tongue, expresses mutated *TP53*, and is a kind gift from Dr. Sophie Pinel. SQ20B and CAL27 cells were maintained at 37 °C with 5% CO_2_ and 90% humidity in Dulbecco’s modified Eagle’s medium (DMEM; PAN Biotech, Aidenbach, Germany) supplemented with 10% fetal calf serum (FCS; Gibco, Thermofisher, Waltham, MA, USA). The human monocytic leukemia THP-1 cell line was a kind gift of Elisabeth Martin (UMR1113, Strasbourg), and was maintained at 37 °C with 5% CO_2_ and 90% humidity in Roswell Park Memorial Institute (RPMI) medium supplemented with 10% fetal bovine serum (Gibco, Waltham, MA, USA). The murine oral carcinoma MOC2 cell line was purchased from Kerafast, Inc, (Boston, MA, USA), and was maintained at 37 °C with 5% CO_2_ and 90% humidity in Dulbecco’s modified Eagle’s medium (DMEM; PAN Biotech, Aidenbach, Germany) supplemented with 10% fetal calf serum (FCS; Gibco, Waltham, MA, USA).

### 2.2. In Vitro Cell Survival Analysis

A total of 1 × 10^4^ cells were seeded per well in 96–well microplates (Falcon Multiwell, Thermofisher, Waltham, MA, USA), and different concentrations of cisplatin (Mylan: 0; 0.1; 0.5; 1; 2.5; 7.5; 15; 30; 100 μM), cetuximab (Merck; 5 mg/mL) or PRIMA MET were applied for 48 h in 100 µL of fresh medium. For co-treatments, 2.5 µg/mL of cetuximab and/or 50 µM of prima were added to the different concentrations of cisplatin. MTT assay was performed as previously described by replacing the cisplatin solution with fresh medium supplemented with 5 mg/L MTT (Sigma, Saint-Louis, MO, USA) for 1 h [32]. Cells were lysed in DMSO 100% (100 μL/well). Absorbance measurements were performed at 550 nm with the LB942 Tristar2 Multimode Reader (Berthold Technologies, Bad Wildbad, Germany). The calculation of the IC_50_, IC_75,_ and IC_90_ was performed with the GraphpadPrism V5.02 software (Graphpad, Software, San Diego, CA, USA) using non-linear regression.

### 2.3. Annexin V and PI Flow Cytometry

Cell apoptosis analysis was carried out using FITC-Annexin V and propidium iodide (apoptosis detection kit, BD Biosciences, Franklin Lake, WI, USA) according to the manufacturer’s instructions. Briefly, cells were seeded in 10 cm Petri dishes and treated with cetuximab +/- cisplatin for 24 h or 48 h. Cells were harvested and counted, diluted in annexin buffer (BD Biosciences, Franklin Lake, WI, USA) at a concentration of 1 × 10^6^ cells per 100 µL, and stained with 10 µL of propidium iodide and 5 µL of FITC-Annexin V. After 15 min of incubation, cells were analyzed in flow cytometer on a BD LSRFortessa^TM^ (BD Biosciences, Franklin Lake, WI, USA) after satisfying QC using CST beads. Acquisition and data analyses have been performed using the BD FACSDiva^TM^ Software.

### 2.4. Gene Expression Assays

Gene expression assays on cultured cells were performed by extracting total RNA from pelleted cells using a standard TRIZol procedure (TRI Reagent^®^: TR 118 Molecular Research Center, Cincinnati, OH, USA), according to the manufacturer’s instructions. RNA was retro-transcribed using the High-Capacity cDNA reverse transcription system (Applied Biosystems^TM^, Thermofisher, Waltham, MA, USA), and real-time quantitative PCR was performed using the QuantStudio 3 Real-Time PCR system (Applied Biosystems^TM^, Thermofisher, Waltham, MA, USA). *DDB2*, *FDRX2*, *RPS27L,* and *ZMAT3* expression was measured with pairs of specific primers (see Table S1), and *CXCL9* and *CXCL10* expression was measured with TaqMan probes (see Table S2). The expression of genes of interest was normalized to the expression of *TBP*, used as a reference gene, with the 2^−ΔΔCt^ method.

### 2.5. SDS-PAGE and Western Blot Analysis

Total protein extraction was carried out by homogenizing 1 × 10^6^ cells in 100 µL of 1X Laemmli lysis buffer 6.25 mM Tris (pH 6.8), 1%SDS, 1%DTT, protease, and phosphatase inhibitors, Sigma. A total of 20 or 30 μg of proteins were resolved by 6%–15% SDS-PAGE (depending on protein molecular weight) according to standard methods. For the analysis of HMGB1 release in the extracellular medium, 40 µL of cells culture supernatant, diluted in SDS-PAGE sample buffer (2X Laemmli lysis buffer, 2X DTT), were resolved by 10% SDS-PAGE. Proteins were detected with primary antibodies raised against cleaved Caspase-3, Calreticulin, EGFR, HMGB1, p63, p53, and p73 (see Table S3 for clones, providers, and concentrations). Depending on the host species, blots were probed with secondary antibodies (1/10,000 anti-mouse IgG-HRP linked antibody, Cell Signaling 7076S; 1/10,000 anti-rabbit IgG-HRP linked antibody, Cell Signaling 7074S) Proteins were visualized with enhanced chemiluminescence using the Clarity™ ECL Western blotting Substrate Bio-Rad reagent, according to the manufacturer instructions. Protein-related signals were acquired on a Pxi Imager (Syngene^®^, Cambridge, United Kingdom). Protein expression (e.g., cleaved Caspase-3, CRT, and HMGB1) was quantified by measuring the SDS-PAGE gel bands using the ImageJ software. In short, and according to the manufacturer’s recommendations, a box was drawn in lanes around gel-band signals using the rectangle tool, making sure to include some of the empty gel between lanes and white space outside of the band. The same box was used for all gel-bands on the same blot. Signal acquisition of pixels was converted into peaks by the ImageJ software, and the area of each peak (which correlates with the gel-band signal intensity) was recovered. Each recovered value was normalized to their respective loading control in the same lane (cell “housekeeping” proteins (actin or GADPH) in the case of cleaved caspase-3 or intracellular HMGB1; cell-culture medium Bovine Serum Albumin (BSA) in the case of extracellular expression of released HMGB1). Finally, the protein of interest to loading control ratios were further normalized by setting the value of this ratio to 1 in the negative control (e.g., non-treated cells). Enrichment of CRT in the membrane protein fraction was evaluated by normalizing the quantification value in a given condition to the quantification value from the same condition in the input.

### 2.6. Biotinylation and Immunoprecipitation of Cell Surface Proteins

Biotinylation and recovery of cell surface proteins were performed with a method adapted from Gottardi et al. [33], Hanwell et al., and T. Panaretakis et al. [34]. Briefly, cells were grown and treated in 10 cm Petri dishes, were washed three times with ice-cold PBS-Ca^2+−^Mg^2+^ (PBS with 0.1 mM CaCl_2_ and 1 mM MgCl_2_), and placed on ice. Membrane proteins were then biotinylated with 1.25 mg/mL NHS-SS-biotin (Pierce) freshly diluted in biotinylation buffer (10 mM triethanolamine, 2 mM CaCl_2_, 150 mM NaCl, pH7.5) for 30 min incubation at 4 °C under gentle agitation. Cells were then rinsed and washed in with PBS-Ca^2+−^Mg^2+^-glycine (100 mM) buffer at 4 °C to quench unreacted biotin. Cells were further rinsed three times with PBS-Ca^2+−^Mg^2+^, scraped in cold PBS, and pelleted by centrifugation (800 rpm at 4 °C) and total protein was harvested for 45 min in 500 μL of lysis buffer (1%Triton X-100, 150 mM NaCl, 5 mM EDTA, 50 mM Tris pH7.5) containing protease inhibitors. A total of 500 µg of total proteins were incubated for 1 h at 4 °C with packed streptavidin-agarose beads to bind to biotinylated proteins. Beads were then pelleted by centrifugation and aliquots of supernatants were sampled to recover unbound, intracellular proteins. Biotinylated proteins (representing membrane proteins) were eluted from the beads by heating to 100 °C for 5 min in an SDS-PAGE sample buffer. Whole-cell proteins (input), the intracellular and membrane protein fractions were further loaded onto a 4–12% gradient gel (Mini protean TGX, Biorad, Marnes-La-Coquette, France) and analyzed by western blot (see above).

### 2.7. Immunofluorescence Staining

CAL27 or MOC2 cells were seeded on coverslips and fixed with PFA 4% for 10 min. In addition, MOC2 cells (but not CAL27 cells) were permeabilized with 0.1% Triton X-100 for 20 min at room temperature. For both cell lines, a saturation of aspecific sites was achieved with 5% Normal Goat Serum for 30 min at room temperature. CAL27 were incubated with anti-CRT antibody (1/400; D3E6 Cell signaling) and MOC2 were incubated with anti-EGFR (1/400; D38B1 Cell signaling) overnight at 4 °C. After 3 washes in 1X PBS, coverslips were further incubated with 1/1000 solutions of goat anti-rabbit-alexa488 (A11034 Invitrogen) secondary antibodies. After 3 washes in 1X PBS, nuclei were labeled with a DAPI (4′,6-diamidino-2-phenylindole) solution (1/20,000) for 5 min, and coverslips were mounted in Calbiochem FluorSave^TM^ reagent (Merck Millipore, Darmstadt, Germany). Pictures were taken with a Zeiss Axio Imager M2-Apotome2 fluorescence microscope.

### 2.8. Generation of hEGFR-MOC2 Clones

MOC2 cells were transduced with lentiviral particles carrying the p-BABE-puro-hEGFR (gift from Dr Di Fiore Pier Paolo) or the empty vectors. Cells were selected with puromycin (8 μg/mL) and checked for ectopic human EGFR expression by western blot. The clone selection was realized by high dilution and seeding of isolated cells. Every clone was then tested by western blot and immunocytofluorecence for the expression of human EGFR.

### 2.9. Vaccination Assay

All animal experiments were approved by the local ethic comity and the French Ministry of Agriculture under the permit APAFiS#29181. C57BL/6 mice (Janvier labs, Le Genest-Saint-Isle, France) were housed in the certified animal facility (#H-67-482-21). Female mice (8 weeks old) were inoculated in the right flank with 5 × 10^5^ hEGFR-MOC2-C1 cells (treated ex vivo with cisplatin, cetuximab or cotreatment cisplatin plus cetuximab) in 100 µL of DMEM. The injection of hEGFR-MOC2-C1 cells killed by three successive freeze/thaw cycles was used a non-immunogenic cell death inducer (negative control). After 7 days, a second challenge was carried out by injecting 5 × 10^5^ hEGFR-MOC2-C1 cells in 100 µL of DMEM in the contralateral flank of the same mice. A minimum of 11 animals in the negative control group (Freeze/thaw cycle group) and a maximum of 12 animals in all other treatment groups were used. Tumor growth was monitored over time by measuring the two dimensions with a caliper. Tumor-free survival was analyzed with Kaplan-Meier survival analysis (see below) and log-rank post-test. Tumors were dissected from mice for further investigation of marker expression by immunofluorescence (see below).

### 2.10. Statistical Analysis

MTT assays results were analyzed with a Mann-Whiney test. For all other data sets, the, the hypothesis of normality (d’Agostino and Pearson test; Shapiro-Wilk test) and homogeneity of variances (Levene test for equality of variances) of data sets were analyzed. If the sample did not meet at least one of these conditions, then a non-parametric test was used (Kruskal Wallis with Dunn post-test). Otherwise, parametric tests were used (Student *t*-test; Anova and Tuckey post-test). The tumor-free survival of mice challenged with a hEGFR-MOC2-C1 cell injection was evaluated with Kaplan-Meier survival analysis (see below) and log-rank post-test. Statistical tests were performed using GraphPad Prism 8. For all analyzes, statistical significance is represented in graphs using asterisks: * *p* < 0.05; ** *p* < 0.001; *** *p* < 0.0001.

## 3. Results

### 3.1. Cetuximab and Cisplatin Inhibit HNSCC Cell Cycle and Trigger Apoptotic Cell Death

**Figure 1:**
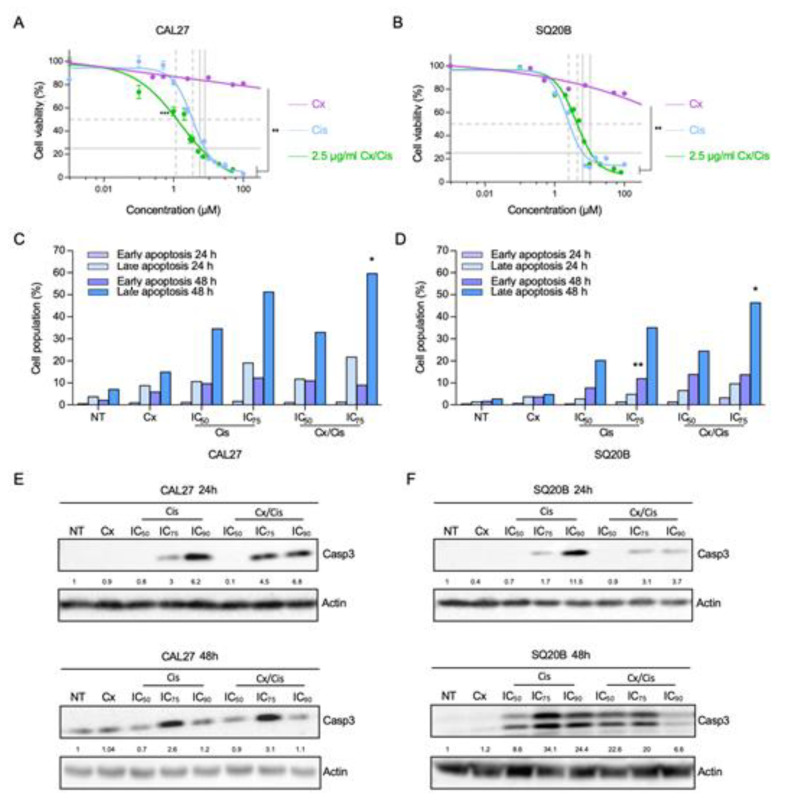
(**A**,**B**). Analysis of CAL27 (**A**) and SQ20B (**B**) cells survival upon treatment with increasing concentrations of cetuximab (Cx; purple line), of cisplatin (Cis; blue line) and of cisplatin +2.5 µg/mL cetuximab (green line), using a MTT-based assay. Mean values from three independent experiments are plotted as sigmoid curves and the cisplatin IC_50_ (dotted grey lines) and IC_75_ (plain grey lines) in the Cis and Cx+Cis conditions are shown. Mann-Whitney *p*-values: * *p* < 0.05; ** *p* < 0.01; *** *p* < 0.001. (**C**,**D**). Annexin V/Propidium Iodide apoptosis assay of CAL27 (**C**) and SQ20B (**D**) cells treated 24 h and 48 h with cetuximab (Cx), cisplatin (Cis) at the IC_50_ and IC_75_ and the Cx/Cis combination. The histograms show the mean number of percentages of early (annexin V-positive, Propidium Iodide-negative; purple) and late (annexin V-positive, Propidium Iodide-positive; purple) cells values from two independent experiments, after 24 h (light colors) and 48 h (dark colors) of treatment. Each treatment condition was compared to its respective non-treated control: ANOVA and Tuckey post-test *p*-values: * *p* < 0.05; ** *p* < 0.01. (**E**,**F**). Western blot analysis of cleaved caspase-3 (Casp3) expression in whole protein extracts from CAL27 (**E**) and SQ20B (**F**) cells treated with cetuximab (Cx), cisplatin (Cis) at the IC_50_, IC_75_ and IC_90_, and the Cx/Cis combination for 24 h (upper panels) and 48 h (lower panels). Signals were quantified respectively to the actin loading control and normalized with respect to the non-treated control (quantification results are shown). The blots shown here are representative examples of three independent experiments. Additional independent biological replicates are shown in supporting documents.

In order to investigate the underlying mechanisms of the cotreatment with cetuximab and cisplatin used in the EXTREME protocol, we performed cell viability assays using CAL27 and SQ20B cell lines. Cells were exposed to increasing concentrations of cetuximab or cisplatin and the cell survival rate was measured with MTT and used to determine the drugs IC_50_, IC_75_ and IC_90_ (i.e., concentrations that result in 50%, 75% and 90% of the maximal drug effect, respectively). Survival curves obtained upon cetuximab treatment showed a maximal drop to ~70% of surviving cells in both cell lines (Figure 1A,B). This maximum effect was reached at 2.5 µg/mL, and higher concentrations of cetuximab did not yield more biological effect. Therefore, we performed MTT assays using a co-treatment with cetuximab (2.5 µg/mL) and increasing concentrations of cisplatin. As expected, the addition of cisplatin to cetuximab was more cytotoxic than cetuximab alone (Mann-Whitney *p* < 0.01 in CAL27 cells and *p* < 0.05 in SQ20B cells). The cotreatment appeared slightly more cytotoxic on CAL27 cells compared to cisplatin alone than in SQ20B cells (IC_50_ = 2.4 µM vs. IC_50_ = 3.5 µM, respectively; Figure 1A). Interestingly, in CAL27 cells, cetuximab seems to mostly favor the activity of cisplatin at lower concentrations (Mann-Whitney *p* < 0.001), whereas no difference between cisplatin alone and the combination were observed at higher concentration (for instance, the IC_75_ was similar in both conditions (7.9 µM vs. 7.6 µM; Figure 1A). Intriguingly and in contrast to CAL27 cells, the addition of cetuximab seemed to lower the cytotoxicity of cisplatin in SQ20B cells (IC_50_ = 4.35 µM vs. IC_50_ = 2.9 µM, and IC_75_ = 10 µM vs. IC_75_ = 6.3 µM, respectively; Figure 1B). Consequently, the co-treatment was found to be more effective on CAL27 cells than on SQ20B cells (Figure 1 A,B).

Next, we wanted to determine whether the cetuximab and/or cisplatin treatments impact CAL27 and SQ20B cell survival rates by inducing apoptosis or by inhibiting cell proliferation. To this end, cells were fixed, stained with Propidium Iodide (PI) and analyzed by flow cytometry. Upon 48 h of treatment, an increase of the proportion of cells in the G2 phase at the expanse of the G0/G1 phase was observed in both cell lines, and more particularly at the IC_75_ of cisplatin (with or without cetuximab), suggesting a cell cycle halt in G2 (Figure S1A,B). Then, to discriminate between necrosis and apoptosis, cells were stained with an Annexin V probe (AV) and Propidium Iodide (PI) and analyzed by flow cytometry. The percentage of IP− AV+ (early apoptosis) and IP+ AV+ (late apoptosis) cells was determined by flow cytometry, after 24 h and 48 h of treatment with cetuximab +/− cisplatin. Although a trend for the dose-dependent increase of the proportion of cells in early apoptosis (IP− AV+), no significant difference was detected after 24 h of treatment in both cell lines, except upon treatment of SQ20B cells with the IC_75_ of cisplatin (Figure 1C,D; *p* < 0.01). A stronger trend for a dose-dependent increased rate of cells in late apoptosis (IP+ AV+) was observed in SQ20B cells especially after 48 h (Figure 1D), and in CAL27 cells after 24 h and 48 h of treatment with cisplatin+/-cetuximab. Observed differences only reached statistical significance in CAL27 and SQ20B cells treated with cetuximab and the IC_75_ of cisplatin compared to non-treated cells. In addition, this increase was more important in CAL27 cell line (Figure 1C): for instance, the proportions of CAL27 cells in late apoptosis after 48 h of treatment with the IC_50_ and IC_75_ of cisplatin were ~35% and ~50%, respectively, whereas they reach ~20% and ~35% in SQ20B cells. The combination of cetuximab did not synergize with cisplatin, since the proportions of cells in late apoptosis were of the same order of magnitude in both cell lines.

To confirm the induction of apoptosis upon treatments, we analyzed the level of cleaved caspase-3 by western blot in whole protein extracts harvested from CAL27 and SQ20B cells 24 h and 48 h after treatment. Cleaved caspase-3 was observed in CAL27 and SQ20B cells after 24 h treatment with cisplatin at the IC_75_ and IC_90_, used alone or in combination with cetuximab, in a dose dependent manner (Figure 1E,F, upper panels). After 48 h of treatment of CAL27 cells, the most effective caspase-3 cleavage was observed upon treatment with the IC_75_ of cisplatin +/− cetuximab (Figure 1E, lower panels). At both time points, the cleavage of caspase-3 was similar when cells were treated with cisplatin alone or combined with cetuximab (Figure 1E). In contrast, in SQ20B cells, cetuximab increased the level of cleaved caspase-3 when combined to the IC_50_ of cisplatin for 48 h, whereas it reduced caspase-3 cleavage when combined with higher cisplatin concentration (Figure 1F). In both cell lines and in all experimental conditions, cetuximab used alone did not induce the cleavage of caspase-3 (Figure 1E,F), which is consistent with the low impact of this treatment on cell growth obtained in MTT assays (Figure 1A,B). Hence, altogether, these results show that treatment of HNSCC cells with cisplatin alone or with cetuximab induces caspase-3 cleavage. However, the impact of the addition of cetuximab to cisplatin on caspase3 dependent apoptosis appears to be complex, since it differs and varies in intensity according to several parameters including drug dose, treatment time and cell line.

### 3.2. p53-Independent Induction of Apoptosis

**Figure 2:**
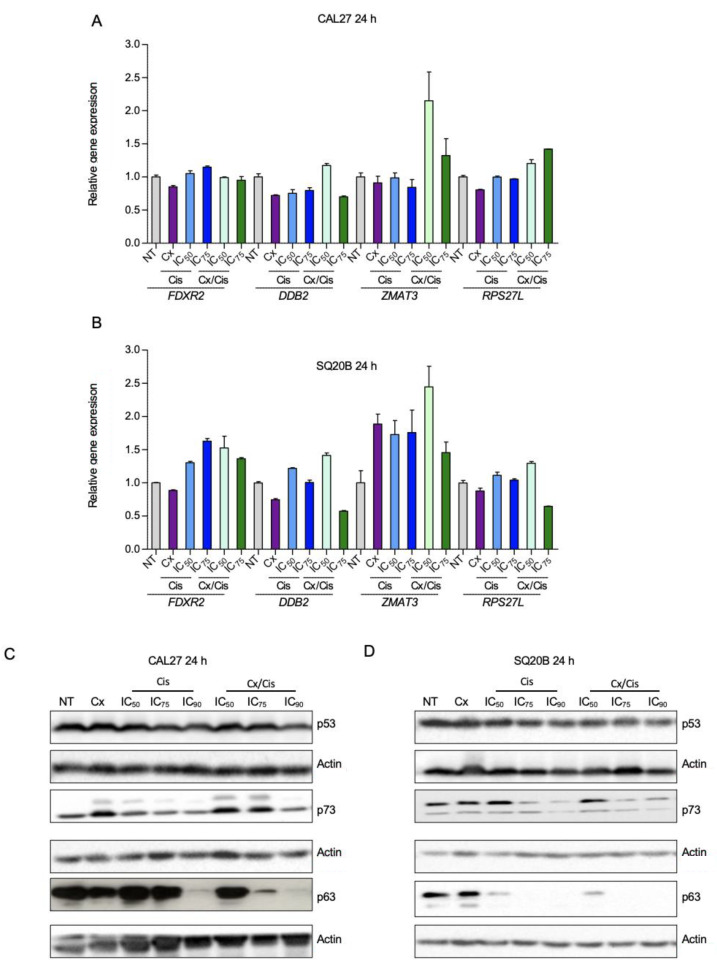
(**A**,**B**). Analysis of the expression of the *FDXR2*, *DDB2*, *ZMAT3* and *RPS27L* genes by RT-qPCR in CAL27 (**A**) and SQ20B (**B**) cells treated for 24 h with cetuximab (Cx), cisplatin (Cis) at the IC_50_ and IC_75_ and the Cx/Cis combination. Data is represented as mean from two independent experiments +SEM. No significant differences were observed between non-treated cells and each treated condition (Kruskal-Wallis and Dunn post-test). (**C**,**D**). Western blot analysis of p53, p73 and p63 expression in whole protein extracts from CAL27 (**C**) and SQ20B (**D**) cells treated with cetuximab (Cx), cisplatin (Cis) at the IC_50_, IC_75_ and IC_90_, and the Cx/Cis combination for 24 h. The blots that are shown are representative examples of three independent experiments.

The p53 family of transcription factors, and especially p53, are well documented mediators of the cytotoxicity induced by DNA damaging drugs, such as cisplatin [35]. Since CAL27 and SQ20B cells were established are from HPV-negative cancers, they both bear a mutated form of p53. CAL27 expresses the mutant H193L p53 that is a gain of function mutant able to interact with YAP [36]. SQ20B harbors a small deletion Tyr126_Lys132del whose impact on p53 activity remains to be established. To understand the contribution of p53 in the effect of the combinatory treatment in CAL27 and SQ20B cells, we first investigated the expression profile of four transcriptional targets of p53 (*FDRX2*, *DDB2*, *RPS27L* and *ZMAT3*) [37] upon treatment with cetuximab+/-cisplatin. No significant impact of the treatments on *DDB2*, *FDRX2*, *RPS27L* and *ZMAT3* gene expression was observed in CAL27 (Figure 2A) and SQ20B cells (Figure 2B), suggesting that p53 is not activated by the treatment in those cells.

We then analyzed the protein level of p53 by western blot. We found the p53 protein to be expressed at high levels in both cell lines, independently of genotoxic treatments (Figure 2C,D). The high expression level and absence of induction by chemotherapy can be explained by the mutation status of p53 in those cells [35].

p53 mutants have deleterious effects on cells and response to chemotherapy by instating new protein interactions, such as with p63 and p73. These interaction blocks p63 and p73 proapoptotic activity, including in response to chemotherapy [38]. Hence, therapeutic strategies with small molecules aiming at restoring p53 function and inhibiting those neo-interactions have been developed [39,40]. Therefore, we wanted to assess whether in CAL27 and SQ20B cells p63 and p73 were expressed and regulated by the treatment, and whether the p53 reactivator PRIMA-MET could favor the impact of the EXTREME protocol in those cells.

p63 and p73 protein levels were analyzed by western blot. Consistently with the fact that the *TP73* gene encodes multiple isoforms (including a full-length isoform with an N-terminal transactivation domain, called TAp73, and a shorter isoform lacking the N-terminal domain, called ΔNp73), two bands were observed when membranes were probed with an anti-p73 antibody [36]. In CAL27 cells, the lower band showed a stronger expression, which was further increased upon cetuximab and cetuximab/cisplatin treatment. The upper band was not detected in non-treated cells, and the expression of this isoform was induced by cetuximab and the cetuximab/cisplatin co-treatment (especially at the IC_50_ and IC_75_), and to a lesser extend upon treatment with cisplatin alone (Figure 2C). Unlike what is observed in CAL27 cells, the most expressed p73 isoform is represented by the upper band in non-treated SQ20B cells, although the lower band was observed. Cisplatin treatments at high doses (i.e., IC_75_; IC_90_), alone or combined with cetuximab, decreased the expression of the upper isoform of p73. The lower isoform was not affected by the treatments (Figure 2D). We also analyzed the expression of the p63 protein, which is expressed as TA and ΔN isoforms, similarly to p73. p63 was found to be expressed in both CAL27 and SQ20B cells, and the p63-related signal appeared as two bands, the upper band being the more expressed. Strikingly, the expression of both isoforms was strongly downregulated upon treatments of CAL27 with the IC_90_ of cisplatin and the cetuximab/cisplatin (IC_75_ and IC_90_) cotreatment (Figure 2C). Similar observations were made in SQ20B cells, where both the IC_75_ and IC_90_ of cisplatin alone or in combination with cetuximab strongly decreased the expression of the p63 protein (Figure 2D).

**Figure 3:**
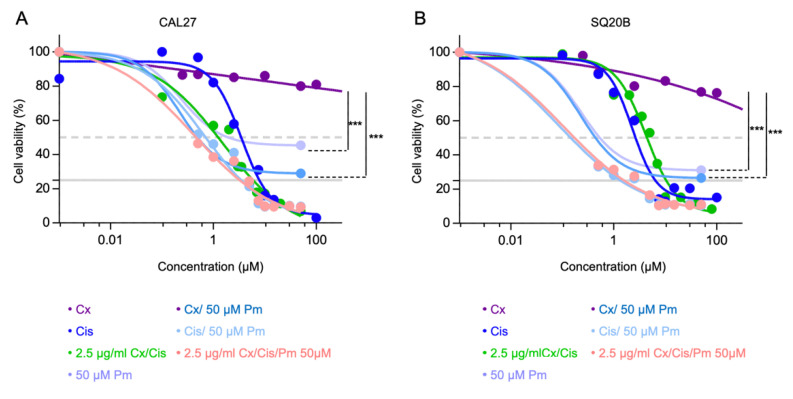
(**A**,**B**). Analysis of CAL27 (**A**) and SQ20B (**B**) cells survival upon treatment with growing concentrations of cetuximab (Cx) +/− 50 µM PRIMA (Pm; dark and light purple lines, respectively), growing concentrations of cisplatin (Cis) +/− 50 µM PRIMA (dark and light blue line, respectively), growing concentrations of cisplatin + 2.5 µg/mL cetuximab +/− 50 µM PRIMA (dark and light green line, respectively), and 50 µM PRIMA alone, using a MTT-based assay. Mean values from 2 independent experiments are plotted as sigmoid curves. The 50% and 25% survival thresholds are shown as a dotted red and plain green lines, respectively. Mann-Whitney *p*-values: *** *p* < 0.001.

To analyze if p53 signaling can be reactivated, SQ20B and CAL27 cell lines were treated with PRIMA MET (50 µM), a p53 reactivator, in addition to cetuximab and/or cisplatin, and cell viability was assessed using a MTT cell survival assay. The treatments with PRIMA +/− cetuximab were found to be more cytotoxic than cetuximab used alone in both cell lines (Mann-Whitney *p* < 0.001). However, no significant increase in cytotoxicity was observed when PRIMA MET was used in combination with cisplatin and/or cetuximab (Figure 3A,B).

Altogether, these results suggest that mutated p53 has no significant impact on the cytotoxicity induced by the cotreatment. Hence, the apoptosis observed in CAL27 and SQ20B cells is independent of p53. In contrast, p63 and p73 isoforms might be involved, but additional investigations are required to precisely identified which isoforms and their respective function.

### 3.3. Danger-Associated Molecular Patterns Are Emitted by HNSCC Cells upon Cetuximab and/or Cisplatin Treatment

**Figure 4:**
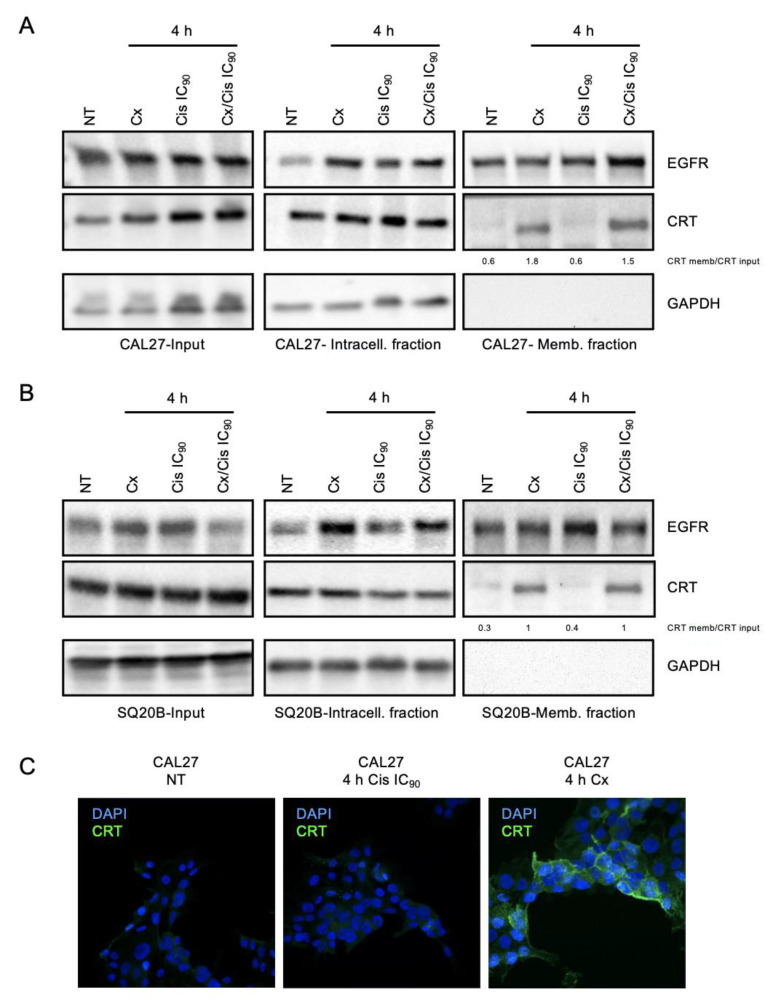
(**A**,**B**). Membrane protein purification and western blot analysis of EGFR, Calreticulin (CRT) and GAPDH expression in the input (left panels), intracellular (middle panels) and extracellular protein fractions of CAL27 (**A**) and SQ20B (**B**) cells treated with cetuximab (Cx), cisplatin (Cis) at the IC_50_, IC_75_ and IC_90_, and the Cx/Cis combination for 4 h. The enrichment of CRT in extracellular fractions is shown. The blots that are shown are representative examples of three independent experiments. Additional independent biological replicates are shown in supporting documents. (**C**). Immunocytofluorescent staining analysis of the expression of CRT in non-treated (NT) CAL27 cells (left panel), and CAL27 treated with cisplatin (Cis) at the IC_90_ (middle panel) or cetuximab (Cx; right panel). Nuclei are stained with DAPI. Magnification: X64.

Using colorectal cancer cell line models, Pozzi and collaborators showed that cetuximab is able to induce an ICD [30]. To explore the capacity of cetuximab to induce ICD in HNSCC, we analyzed the emission of several DAMPs by CAL27 and SQ20B cells upon treatment with cetuximab +/− cisplatin. We first assessed the plasma membrane relocalization of the Calreticulin (CRT) chaperone in CAL27 and SQ20B cells treated with cetuximab, cisplatin or the cetuximab/cisplatin co-treatment. First, based on the literature that describes CRT translocation as an early and essential event of ICD, we chose to assess its expression after 4 h of treatment using the detection of membrane proteins using a non-permeant reactive biotin. Cells were incubated with Sulfo-NHS-SS-Biotin to biotinylate plasma membrane proteins, and streptavidin beads were used to separate membrane-associated proteins from intracellular proteins. Both protein fractions were resolved with SDS-PAGE and analyzed by western blot. Membranes were probed with an anti-EGFR and an anti-GAPDH antibodies, used as positive and negative controls for the membrane fraction, respectively. Probing the blots with an anti-GAPDH antibody was also used as positive control for both the total input and intracellular fraction. Signals corresponding to the EGFR were observed in the three fractions, whereas no signal corresponding to GAPDH were observed in the membrane fraction, both in CAL27 (Figure 4A) and in SQ20B cells (Figure 4B). Membranes were also probed with a specific anti-CRT antibody, and signals corresponding to CRT in the membrane fraction were normalized to CRT signals in the input. Interestingly, the CRT was found to be ~3 and times ~5 more present in the membrane protein fraction of cells treated with cetuximab or the cetuximab/cisplatin IC_90_ combination, in CAL27 and SQ20B cells, respectively (Figure 4A,B). Considering that no changes in CRT expression are observed in the input, this suggest that CRT is translocated from the endoplasmic reticulum to the plasma membrane upon cetuximab treatment. This observation was further confirmed by an immunocytofluorescence analysis of the expression of CRT in non-permeabilized CAL27 cells (Figure 4C). Altogether, these results show that cetuximab used either alone or in combination with cisplatin induce the plasma membrane translocation of CRT in both SQ20B and CAL27 cells.

**Figure 5:**
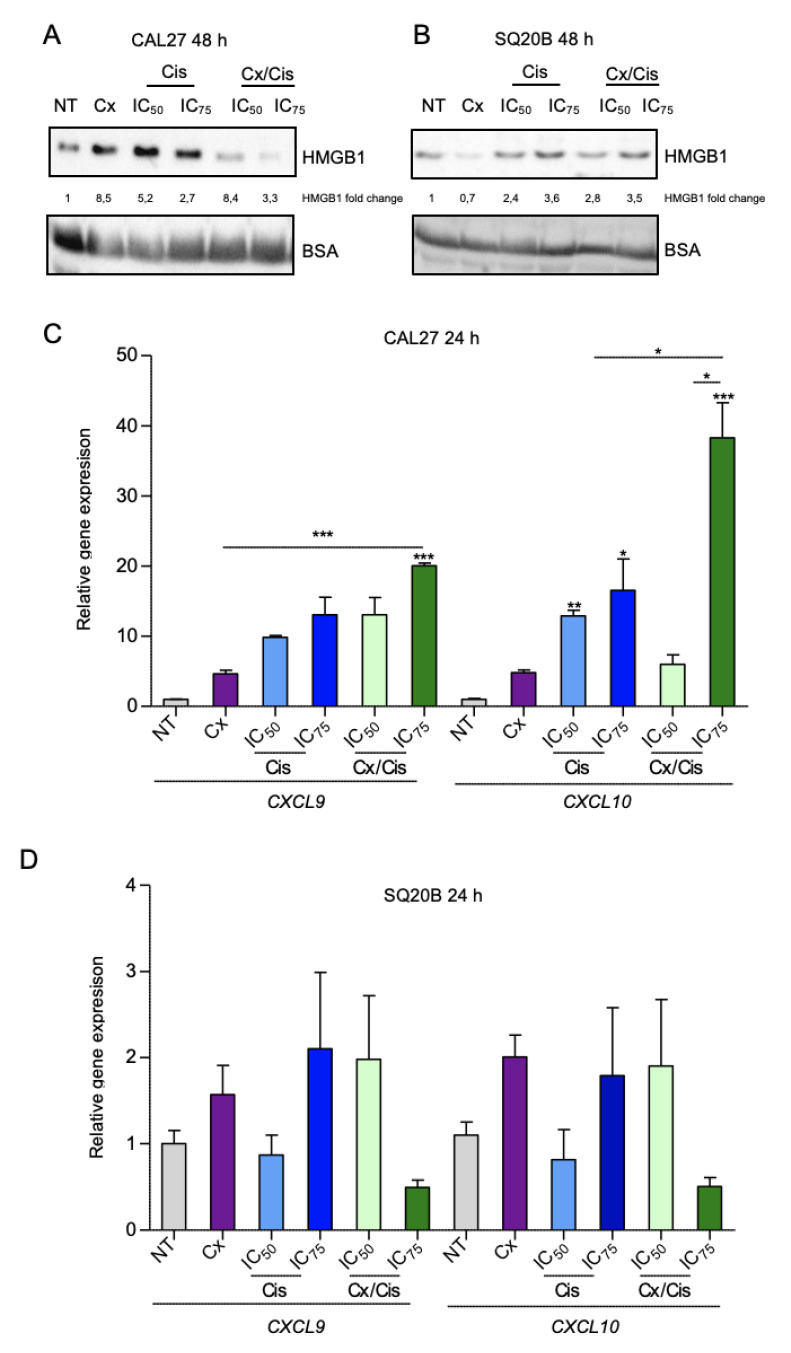
(**A**,**B**) Western blot analysis of the HMGB1 expression in the supernatant from CAL27 (**A**) and SQ20B (**B**) cell cultures harvested 48 h after with cetuximab (Cx), cisplatin (Cis) at the IC_50_ and IC_75_ and the Cx/Cis combination. Signals were quantified respectively to the actin loading control and normalized with respect to the non-treated control (quantification results are shown). The blots that are shown are representative examples of two independent experiments. Additional independent biological replicates are shown in supporting documents. (**C**,**D**). Analysis of the expression of the *CXCL9* and *CXCL10* by RT-qPCR in CAL27 (**C**) and SQ20B (**D**) cells treated for 24 h with cetuximab (Cx), cisplatin (Cis) at the IC_50_ and IC_75_ and the Cx/Cis combination. Data is represented as mean from two independent experiments +SEM. Kruskal-Wallis and Dunn post-test *p*-values: * *p* < 0.05; ** *p* < 0.01; *** *p* < 0.001.

We also investigated the expression of HMGB1, which is known to be released from the nucleus to the extracellular environment at later ICD stages [41]. CAL27 and SQ20B cells were treated with cetuximab +/− cisplatin, and both whole cell proteins and proteins in the cell culture medium were harvested 48 h after treatment, resolved with SDS-PAGE and analyzed by western blot. The level of BSA was used as a loading control of samples of supernatant protein and for normalization. In the CAL27 cell line, HMGB1 was present in the supernatant upon treatment, in all conditions, with the most important signal observed after treatment with cetuximab and with cetuximab/cisplatin IC_50_, with an induction fold compared to the untreated control of 8.5 and 8.4, respectively (Figure 5A). The induction of HMGB1 level was higher upon treatment with the IC_50_ of Cisplatin compared to the IC_75_ (Figure 5A). On the contrary, HMGB1 level in the supernatant of SQ20B cells was only triggered with treatments involving cisplatin and, in a concentration-dependent manner (i.e., cisplatin alone and the cisplatin/cetuximab with similar expression patterns; Figure 5B).

Finally, we analyzed the expression of type I interferon-responsive cytokines using RT-qPCR 24 h after treatment, and namely *CXCL9* and *CXCL10*, that are known to be upregulated by type I interferons [42]. In CAL27 cells, the expression of both *CXCL9* and *CXCL10* was strongly upregulated: a significant ~10-fold induction of *CXCL10* (compared to non-treated control) was observed upon treatment with cisplatin alone. Treatment of CAL27 cells with the cetuximab/cisplatin IC_75_ combination triggered a ~20-fold and ~45-fold induction of *CXCL9* and *CXCL10*, respectively (Figure 5C). Conversely, no statistically significant impact of the treatments on *CXCL9* and *CXCL10* expression was observed in SQ20B cells (Figure 5D). However, the biological impact on treatments of these cytokine gene expression appears more complex. Indeed, a gene expression assay was also carried out 48 h after treatment, and we observed that *CXCL9* and *CXCL10* are downregulated in both cell lines upon cisplatin treatment compared to other conditions (Figure S2B).

Altogether, our observations show that treatment of HNSCC cells with cetuximab induced the emission of DAMPs in a cell-dependent manner: CAL27 cells treated with cetuximab alone or combined with cisplatin appear to be more prone to the emission of DAMPs (CRT plasma membrane translocation; HMGB1 release; induction of type I interferon response) than SQ20B cells. Interestingly, platinum-based chemotherapy alone does not trigger CRT exposure and appears to repress *CXCL9* and *CXCL10* expression 48 after treatment.

### 3.4. Cetuximab +/− Cisplatin Trigger DAMPs Emission in Murine Head and Neck Carcinoma Cells

**Figure 6:**
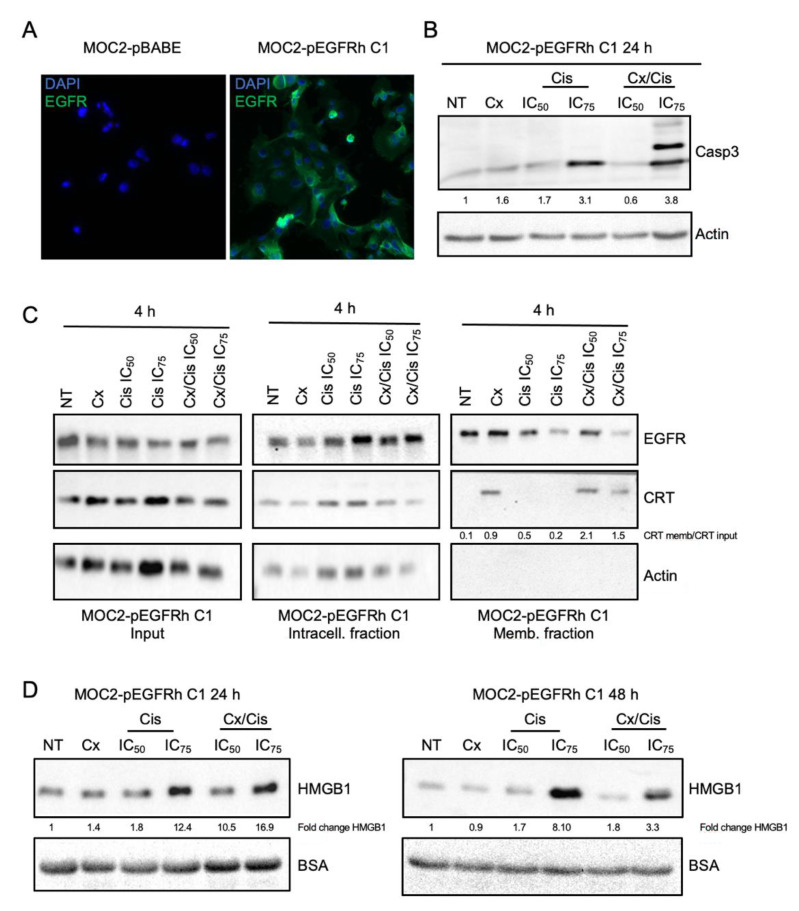
(**A**). Immunocytofluorescent staining analysis of the expression of EGFR in MOC2 cells that were stably transfected with the empty pBABE (left micrograph) and pBABE-hEGFR (right micrograph, MOC2-phEGFR C1 clone) expression plasmid. Nuclei are stained with DAPI. X40. (**B**). Western blot analysis of cleaved caspase-3 (Casp3) expression in whole protein extracts from MOC2-phEGFR C1 cells treated with cetuximab (Cx), cisplatin (Cis) at the IC_50_ and IC_75_, and the Cx/Cis combination for 24 h. Signals were quantified respectively to the actin loading control and normalized with respect to the non-treated control (quantification results are shown) (**C**). Membrane protein purification and western blot analysis of EGFR, Calreticulin (CRT) and Actin expression in the input (left panels), intracellular (middle panels) and extracellular protein fractions of MOC2-phEGFR C1 cells treated with cetuximab (Cx), cisplatin (Cis) at the IC_50_ and IC_75_, and the Cx/Cis combination for 4 h. The enrichment of CRT in extracellular fractions is shown. (**D**). Western blot analysis of the HMGB1 expression in the supernatant from MOC2-phEGFR C1 cell cultures harvested 24 h (left panels) and 48 h (right panels) after with cetuximab (Cx), cisplatin (Cis) at the IC_50_ and IC_75_ and the Cx/Cis combination. All blots shown in this figure are representative examples of three independent experiments. Additional independent biological replicates are shown in supporting documents.

To confirm that the cetuximab +/− cisplatin treatment modifies the immunogenicity of HNSCC cells, anti-cancer prophylactic immunizations of mouse syngeneic models were carried out. To this end, we used the MOC2 mouse head and neck carcinoma cell line to generate a stable murine cell line expressing the human EGFR (hEGFR). After retroviral transduction of MOC2 cells using an hEGFR expression plasmid and selection on puromycin, several clones were obtained, one of which (MOC2-phEGFR-C1) was found to express the hEGFR protein (Figure S3). An immunocytofluorescent staining of hEGFR of MOC2-phEGFR-C1 cells showed that the expression of hEGFR is homogeneous in the cell population (Figure 6A). Consistently with what was observed in CAL27 and SQ20B cells, treatment with cisplatin alone or in combination with cetuximab induced caspase-3 cleavage, 24 h (Figure 6B) and 48 h (Figure S4A) after treatment.

Interestingly, the same treatments were also found to trigger the relocalization of CRT to the plasma membrane (Figure 6C). However, unlike CAL27 and SQ20B, the cetuximab/cisplatin co-treatment was able to induce a more robust expression of CRT at the plasma membrane of MOC-2-phEGFR-C1 cells compared to treatment with cetuximab alone (Figure 6C, right panels). Finally, we observed that HMGB1 was released in the extracellular medium, 24 h, and 48 h after treatment with cisplatin alone or in combination with cetuximab, in a dose-dependent manner (Figure 6D), whereas the expression of intracellular HMBG1 was not affected by treatments (Figure S4B).

### 3.5. Induction of Bona Fide ICD by Cetuximab +/− IC_50_ of Cisplatin

**Figure 7:**
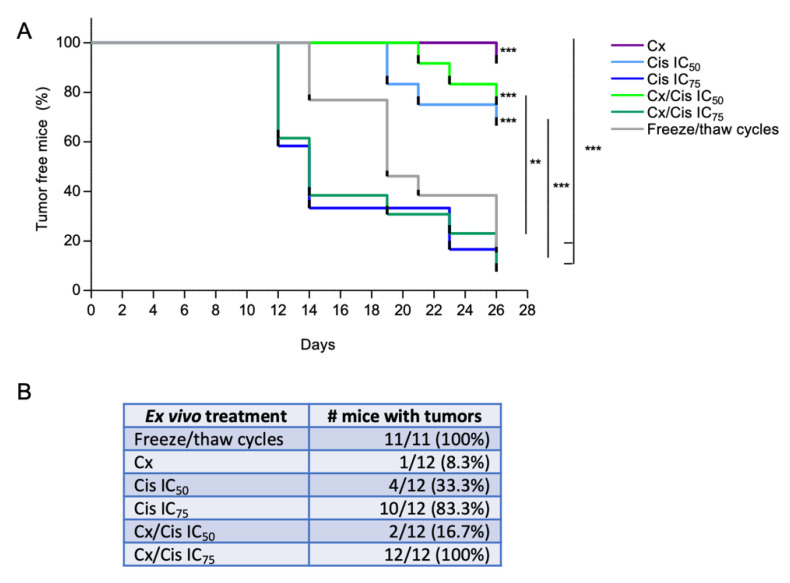
(**A**). Kaplan-Meier analysis of the tumor-free survival of MOC2-hEGFR syngeneic mice models vaccinated with ex vivo treated cells. Treatments include cetuximab (Cx), the IC_50_ and IC_75_ of cisplatin (Cis IC_50_; Cis IC_75_), the cetuximab and cisplatin combination (Cx/Cis IC_50_; Cx/Cis IC_75_) and cells killed by freeze/thaw cycles. Kaplan-Meier *p*-values: ** *p* < 0.01; *** *p* < 0.001. (**B**). Analysis of the number of tumor-free mice. The ex vivo treatment arms, number of treated mice that grew tumors/total number of mice and percentage, and the mean volume of tumors are shown.

To assess whether the treatment of MOC2-phEGFR-C1 cells by cetuximab and/or cisplatin was able to induce an anti-tumor immune response in vivo, MOC2-phEGFR-C1 cells were first treated ex vivo, and dead cells were injected in the right flank of immunocompetent C57BL/6 mice. The same mice were then challenged seven days after the first immunization, by injecting non-treated living MOC2-phEGFR-C1 cells on the left flank, and the mice progression-free survival was evaluated. An injection of MOC2-phEGFR-C1 cells killed by three consecutive freeze/thaw cycles was used as a non-immunogenic cell death control. Expectedly, tumor progression was observed within 26 days in 11/11 mice that were injected with MOC2-phEGFR-C1 cells killed by freeze/thaw cycles (Figure 7A,B). A similar observation was made for mice in the IC_75_ cisplatin (tumor growth in 10/12 mice) and cetuximab + IC_75_ cisplatin (tumor growth in 12/12 mice) treatment groups. Strikingly, the injection of cells treated with cetuximab, the IC_50_ of cisplatin, or the combination was able to prevent tumor progression, which was observed in only 1/12, 4/12, and 2/12 mice, respectively (Figure 7A,B). Thus, these observations suggest that the injection of MOC2-phEGFR-C1 cells treated with cetuximab and/or the IC_50_ of cisplatin provides protection against a subsequent tumor challenge.

## 4. Discussion

HNSCC is a particularly deadly cancer, with approximately 400,000 cancer-related deaths in 2018 [2]. Cetuximab, which targets EGFR, is the only approved targeted therapy for the management of locally advanced R/M HNSCC. However, Cetuximab is not used as a monotherapy, but is associated with either radiotherapy [1] or platinum-based chemotherapy (EXTREME regimen) [7]. To the best of our knowledge, the molecular mechanisms underlying the cytotoxicity of the EXTREME regimen have not been investigated in detail in HNSCC.

Here we showed that the cetuximab/cisplatin combined treatment displays higher toxicity than both treatments provided alone in the CAL27 cell line. These in vitro observations are consistent with clinical trials, where the EXTREME protocol results in a 3-month increase in the overall survival of patients with HNSCC compared to monotherapies [6]. In addition, similar observations were also made in nasopharyngeal cancer [43] and in colon cancer models [44]. More specifically, the cetuximab/cisplatin combination was shown to block the EGFR pathway via the inhibition of EGRF and ERK phosphorylation in the HCT116 and SW480 human colon cancer cell lines [44]. ERK is a kinase of the MAP Kinase pathway downstream of the EGFR, whose activation leads to cell proliferation. Most interestingly, ERK inhibition seems to be critical for the synergic effect of the cetuximab/cisplatin treatment, since it plays a role in the resistance to cetuximab, which can be overcome by a dual blockade of EGFR and HER3 [44,45]. However, no improved cytotoxicity of the cetuximab/cisplatin was observed in SQ20B cells. We previously have shown that the high basal and cetuximab-induced expression of the Hypoxia Inducible Factor-2α transcription factor in SQ20B cells is responsible for resistance to EGFR-blockage [8]. This could explain the lack of benefit from treatment with cetuximab. In addition, additional unidentified molecular disorders in SQ20B cells could account for the absence of synergy between cisplatin and cetuximab. Thus, the differential cytotoxicity of the cisplatin/cetuximab combination in CAL27 and SQ20B cell lines illustrates that different genetic and molecular backgrounds are likely to dictate cell response to the therapy. Key molecular differences between the two cell lines that are likely to include the *TP53* mutational status (CAL27 and SQ20B harbor different *TP53* gene mutations, of unknown functional consequences), which might influence the induction of apoptosis upon genotoxic stress, as well as the basal and induced expression level of the Hypoxia Inducible Factor-2 (HIF-2) transcription factor (CAL27 express the lower basal level of HIF-2 that are unaffected by cetuximab treatment, whereas SQ20B expresses higher levels of HIF-2, that is upregulated upon cetuximab treatment), which is responsible for resistance to EGFR blockade in SQ20B cells [8]. These observations also highlight that the response of patients to the EXTREME protocol is likely to vary depending on tumor-specific molecular signatures.

We further analyzed the impact of the EXTREME regimen on both cell cycle deregulation and the induction of apoptotic cell death, which both contribute to cell growth, by using specific markers (iodide propidium/annexin V; cleaved caspase-3). Our observations suggest that the co-treatment induces both a cell cycle arrest in the G2 phase, as well as apoptotic cell death. More particularly, we observed that cisplatin +/− cetuximab triggered caspase-3 cleavage in both CAL27 and SQ20B cells, as well as in MOC2-phEGFR C1 cells. The induction of caspase-3 cleavage and upon cetuximab/cisplatin cotreatment was previously reported in the HCT116 and SW480 colon cancer cell lines, where both molecules seem to synergize [44]. However, the benefit of adding cetuximab to cisplatin with respect to caspase-3 cleavage and the extent of the synergy appears to depend on drug concentration, treatment timing, and treated cell line. Indeed, a mild synergy was observed in CAL27 and SQ20B after 24 h of treatment with the combination of the IC_50_ of cisplatin and cetuximab, whereas a stronger synergy was observed only in SQ20B cells after 48 h of treatment with cetuximab and cisplatin at the IC_75_. Strikingly, the combination using high doses of cisplatin performed more poorly in SQ20B cells than drugs used alone. This suggests that SQ20B might undergo caspase-3-independent cell death upon treatment with high doses of cisplatin. This also highlights that cell response to genotoxic stress is likely to vary according to both the dose and the duration of treatment, with the induction of different signaling pathways. The level of activation of these pathways is probably dependent on the molecular and genetic background of each cell line. Further detailed analyses of these mechanisms are required to shed more light on the molecular basis of these phenomenon.

The p53 family of transcription factors is involved in DNA damage repair and apoptosis induction upon platinum-derived compound treatment [46]. Importantly, the *TP53* gene is mutated in >80% of HNSCC and plays a major role in tumor initiation and progression and resistance to platinum-based chemotherapy [47]. Interestingly, the role of p53 in the modulation of the tumor immune microenvironment and response to immunotherapy has recently emerged in the literature [48,49]. In addition, it was shown the pharmacological reactivation of p53 by Nutlin-3a induces the release of DAMPs and the activation of ICD [50]. Therefore, in order to gain further insight into the molecular mechanisms associated with the induction of apoptosis by the EXTREME protocol, and its potential correlation to the induction of ICD, we analyzed the expression of the three members of the p53 family (i.e., p53, p63, and p73). The CAL27 and SQ20B cell lines have mutated p53 and consequently, non-treated cells expressed a high level of the p53 protein. Our western blot analysis did not show any modification of the p53 protein expression level upon treatment with cetuximab and cisplatin, alone or combined, and the expression of known p53 target genes was not induced upon treatment, suggesting that p53 might not be involved in the apoptosis of CAL27 and SQ20B cells. The crosstalk between the p53 and the EGFR signaling pathway, and its consequence on therapeutic EGFR blockade has been shown in other cancer models. For instance, both wild-type and mutant p53 have been proposed to regulate *EGFR* transcription [51,52], and inhibition of p53 results in EGFR downregulation in prostate cancer cells [53]. Resistance to cetuximab has been correlated to a loss of p53 expression and an increase of p-ERK expression in H226 lung cancer cells and SCC6 HSNCC [54]. Consistently, p53 was shown to functionally impact the response of H226 cells to EGFR blockade [54], and the restoration of p53 function in p53 null prostate cancer cells stimulates EGFR and Akt phosphorylation and restores sensitivity to cetuximab [55]. We attempted to restore p53 function by treating SQ20B and CAL27 cells with the PRIMA MET reactivator but did not observe any consequences in terms of cell response to cetuximab and/or cisplatin. We cannot rule out that p53 reactivation was not achieved in our hands due to the nature of the mutations present in both cell lines, and additional functional studies about the role of p53 in the response of HNSCC cells to the EXTREME protocol are required.

The *TP73* and *TP63* genes encode two isoforms: one longer isoform called TA, which contains the N-terminal translational domain and displays pro-apoptotic function, and one shorter isoform called ΔN, which lacks the translational domain and has oncogenic activity [56]. Very scarce data is available on the expression and role of p73 in HNSCC. It has been reported to be expressed at lower levels compared to the other member of the p53 family (i.e., p53 and p63) [57]. Two bands were observed in a western blot analysis of p73 and p63 expression in whole protein extracts from CAL27 and SQ20B cells. ΔNp73, which could correspond to the lower band, seems to be the dominant p73 isoform in CAL27 cells. However, a higher band, which could correspond to TAp73, is induced by cetuximab +/− cisplatin at the IC_50_. This might suggest a modification of the ratio of the p73 isoforms in CAL27 cells, with the induction of the expression of the pro-apoptotic TAp73 isoform (possibly the upper band) upon treatment. Interestingly, a modification of the TA/ΔNp73 (possible the upper and lower band, respectively) ratio is also observed in SQ20B cells, with a reduced expression of the TAp73 isoform upon treatment with higher doses of cisplatin (IC_75_ and IC_90_). However, formal identification of the bands is required to confirm this hypothesis. TAp73 can induce apoptosis by indirectly regulating p53 target genes [11]. Most interestingly, EGFR signaling blockade by cetuximab has been shown to inhibit AKT and ERK, thus relieving p73 inhibition and subsequent transactivation of PUMA and the induction of mitochondrial stress-related apoptosis [58]. ΔNp63 is the dominant p63 isoform in HNSCC and is known to play a critical role in carcinogenesis and tumor cell survival [57,59]. In our hands, the expression of ΔNp63 was downregulated by high doses of cisplatin, and treatment with cetuximab increased this effect. The effect of the cisplatin +/− cetuximab treatment was found to be more important in SQ20B cells, where it resulted in total inhibition of the expression of ΔNp63. Importantly, ΔNp63 is known to inhibit p73-related apoptosis on HNSCC cells through direct physical interaction and direct binding on response elements if the promoter of *PUMA* [59,60]. Our observations are therefore consistent with the downregulation of ΔNp63 and subsequently lifted inhibition on p73 expression and/or activity upon treatment with cetuximab+/−cisplatin, which might participate in the induction of apoptotic cell death by the EXTREME protocol. Additional functional data are required to test this hypothesis.

In addition to the role that the EXTREME protocol might play in apoptosis, we also investigated whether it was able to induce ICD. Indeed, Pozzi and colleagues have shown that cetuximab is capable to trigger the emission of DAMPs as well as the activation of anti-tumor immunity in colon cancer cells [30]. Consistently with their findings, we found that cetuximab alone or in combination with cisplatin-induced the relocalization of CRT to the plasma membrane in both SQ20B and CAL27 cell lines as early as 4 h after treatment. The CRT chaperone is known to translocate from the ER to the cell surface early during ICD [61], where it is recognized by the CD91 receptor on antigen-presenting (APC) cells and acts as an “eat me” signal [27]. Consistently with the fact that cisplatin is not an ICD-inducer [62], we did not observe CRT relocalization upon treatment with cisplatin. The extracellular release of HMGB1 was triggered upon all treatments in CAL27 cells, and upon treatment with the combination only, in a dose-dependent manner, in SQ20B cells. The extracellular release of the chromatin-associated HMGB1 protein has an immunomodulatory function, including cytokine activity and pro-inflammatory activity, that depends on its oxidation state [63]. In the frame of ICD, HMGB1 is recognized by TLR4 and induces dendritic cell activation, increasing phagocytosis of tumors antigen liberated by dying cells [41]. Although, the mechanism allowing the release of HMGB1 is unknown [27], we observed a correlation between the presence of HMGB1 in the extracellular medium and the percentage of CAL27 and SQ20B cells in late apoptosis 48 h after treatment, suggesting a potential link between the intrinsic sensitivity of HNSCC cells to the EXTREME protocol and HMGB1 emission. The production of type I interferons (IFNs) is also a feature of cells undergoing ICD. The secretion of type I IFNs activates signaling pathways through their interaction with the Interferon Alpha and Beta Receptor Subunit 1, in an autocrine and paracrine manner, which ultimately triggers the expression of T-cell chemoattractant chemokines CXC motif ligand CXCL9 and CXCL10 [27,64]. Interestingly, the expression of *CXCL9* and mainly *CXCL10* was found to be increased in the CAL27 cell line, upon treatment by cetuximab and/or cisplatin in a dose-dependent manner. This shows that the EXTREME protocol can trigger the secretion of immunomodulatory cytokines in HNSCC cells, although our observation suggests that this effect is likely to be cell-dependent. In conclusion, and consistently with what was shown by Pozzi and collaborators in colon cancer cells [30], we found that cetuximab, alone or with cisplatin, can trigger the emission of several DAMPs (including CRT, HMGB1, and type I IFN response) in HNSCC cell lines, according to different patterns and/or intensity. Further studies will be required to assess whether this heterogeneity of response is also found in human tumors, as well as the relevance it might have with response to the treatment and patient outcome.

In order to validate the immunogenic nature of the treatment of HNSCC cells by cetuximab +/− platinum-based chemotherapy, we performed a vaccination assay using immunocompetent syngeneic models, which is considered to be the gold standard to demonstrate ICD in vivo [26]. To this end, a MOC2 cell line stably expressing the human EGFR was generated. The parental MOC2 cells were derived from a tumor in the oral cavity of a C57BL/6 mouse, and generated aggressive tumors within an immunosuppressive environment [65]. Similar to what was observed in CAL27 and SQ20B cells, MOC2-phEGFR-C1 cells incubated with cisplatin and/or the cisplatin/cetuximab combination displayed apoptosis features (caspase-3 cleavage), and the plasma membrane translocation of CRT and extracellular release of HMGB1. Interestingly, ex vivo treatment of MOC2-phEGFR-C1 cells with either cetuximab, the IC_50_ of cisplatin, or their combination provided mice an anti-tumor protection against a second tumor challenge, whereas treatment with the IC_75_ of cisplatin +/− cetuximab did not. Our results suggest therefore that cetuximab can induce ICD of HNSCC cells, which is consistent with previous observations on murine lung cancer cell line [66] and on murine colon cell line expressing a human EGFR [30]. Surprisingly, we found the immunization effect provided by cisplatin treatment to be depending on drug concentration and does not correlate with the dose-dependent effect we observed in vitro on the emission of DAMPs. This could be explained by the fact that caspase-3 cleaved is induced at higher levels upon treatment with the IC_75_ of cisplatin. Indeed, the activation of caspase-3 stimulates the exposure of phosphatidylserine (PS) on the outer leaflet of cells’ plasma membrane. The recognition of PS by specific PS receptors stimulates the uptake of apoptotic corpses by phagocytes of the immune system together while delivering an anti-inflammatory signal (see [67] and references therein). In addition, caspase-3 is known to stimulate the expression of prostaglandin E2, which has immunosuppressive functions [67]. Finally, caspase-3 inhibits signals known as DAMPs, including the IL-33 cytokine as well as intracellular signals that lead to the expression of type I IFNs [67]. Thus, our observations suggest that the induction of the immunogenicity of cancer cells upon cytotoxic treatment could correlate inversely with the intensity of the induction of caspase-3-related cell death, through the number of cells that undergo apoptosis and/or the level of induction of caspase-3 protein.

The lack of correlation between cisplatin concentrations and the immunogenic effect of the treatment in vivo is consistent with clinical data showing that cytotoxic anti-cancer drugs delivered at lower doses with metronomic treatment schedules rather than administrated at their maximum tolerated dose influence the infiltration of treated tumors with immune cells (for review see [68] and references therein): indeed, drugs used at their maximum tolerated dose in order to provide a high cytotoxic effect, whereas lower suboptimal doses have been shown to stimulate an anti-tumor effect through the stimulation of the immune system. Interestingly, the intra-tumor deliverance of nano-doses of conventional chemotherapeutic drugs has also been reported to make up the tumor immune landscape [69]. Altogether, this highlights the relative importance of the drug-related induction of apoptotic cell death vs. the emission of ICD mediators, which is more desirable to stimulate the immune system and possibly synergize with immunotherapies. Thus, there might be a subtle balance between treatment-induced stress, which could result in the improvement of cancer cell immunogenicity, and treatment-induced cell death, which potentially hinders cancer cell immunogenicity via immunomodulatory signals.

In conclusion, we have shown that cetuximab (either alone or in combination with cisplatin) is able to enhance murine and human HNSCC cells’ immunogenicity through the exposure of CRT, which is known to provide a strong “eat-me” signal to phagocytes of the immune system. However, the impact of cetuximab alone on the release of HMGB1 varies in a cell-dependent manner, while cisplatin (alone or in combination with cetuximab) appears to stimulate HMGB1 release from apoptotic cells. Finally, cisplatin +/− cetuximab appears to trigger a type I IFN response that elicits the expression of CXCL9 and CXCL10 in a cell-line-dependent manner. Interestingly, only ex vivo cell treatment conditions that allow the release of DAMPs and moderate cleavage of caspase-3 (i.e., cetuximab and/or cisplatin at the IC_50_) appear to be able to elicit an anti-tumor immune response in immunocompetent animals. Further studies are warranted to evaluate whether variations of the EXTREME protocol including the dose of cisplatin are able to trigger ICD and provide a similar effect, and to what extent this can be synergistic with immunotherapies in HNSCC patients.

## Data Availability

Not applicable.

## Supplementary Materials

The following supporting information can be downloaded at: https://www.mdpi.com/article/10.3390/cells11182866/s1: Figure S1. Quantification of the cell cycle distribution data of CAL27 (A) and SQ20B (B) cells treated 24 h (upper histograms) and 48 h (lower histograms) with cetuximab (Cx), cisplatin (Cis) at the IC_50_ and IC_75_ and the Cx/Cis combination. The histograms show the mean number of percentages of cells in S, G2, G1/G0, and early apoptosis (EA; i.e., sub-G1); Figure S2. (A) Western blot analysis of the HMGB1 expression in the supernatant from CAL27 (left panels) and SQ20B (right panels) cell cultures harvested 68 h after with cetuximab (Cx), cisplatin (Cis) at the IC_50_ and IC_75_ and the Cx/Cis combination. Signals were quantified respectively to the actin loading control and normalized with respect to the non-treated control (quantification results are shown). The blots that are shown are re Apresentative examples of three independent experiments. Additional independent biological replicates are shown in supporting documents. (B) Analysis of the expression of *CXCL9* and *CXCL10* by RT-qPCR in CAL27 (left histographs) and SQ20B (right histographs) cells treated for 48 h with cetuximab (Cx), cisplatin (Cis) at the IC_50_ and IC_75_ and the Cx/Cis combination. Data are represented as the mean from one independent experiment +SEM; Figure S3. Western blot analysis of EGFR expression in whole-cell protein extracts from MOC-2 cells transduced with a pBABE-hEGFR expression plasmid after selection of clones on puromycin. EGFR expression is observed in the MOC2-phEGFR C1 clone. The blots that are shown are representative examples of three independent experiments; Figure S4. (A) Western blot analysis of p53 and cleaved caspase-3 (Casp3) expression in whole protein extracts from MOC2-phEGFR C1 cells treated with cetuximab (Cx), cisplatin (Cis) at the IC_50_, IC_75,_ and IC_90_, and the Cx/Cis combination for 24 h (left panels) and 48 h (right panels). Casp3 signals were quantified respectively to the actin loading control and normalized with respect to the non-treated control (quantification results are shown). (B) Western blot analysis of the HMGB1 expression in whole protein extracts from MOC2-phEGFR C1 cell cultures treated for 24 h (left panels) and 48 h (right panels) with cetuximab (Cx), cisplatin (Cis) at the IC_50,_ and IC_75_ and the Cx/Cis combination. Signals were quantified respectively to the actin loading control and normalized with respect to the non-treated control (quantification results are shown). Blots. shown are representative examples of two independent experiments. Additional independent biological replicates are shown in supporting documents; Table S1. List of oligonucleotides primers used for RT-qPCR gene expression assays: Gene names, forward and reverse primer sequences are shown; Table S2. List of TaqMan assays used for RT-qPCR gene expression assays: Gene names and TaqMan gene expression assay ID are shown; Table S3. List of antibodies used for western blot analysis; Protein names, antibody providers and used antibody dilutions are shown.

## References

[1] Johnson D.E., Burtness B., Leemans C.R., Lui V.W.Y., Bauman J.E., Grandis J.R. (2020). Head and Neck Squamous Cell Carcinoma. Nat. Rev. Dis. Primer.

[2] Bray F., Ferlay J., Soerjomataram I., Siegel R.L., Torre L.A., Jemal A. (2018). Global Cancer Statistics 2018: GLOBOCAN Estimates of Incidence and Mortality Worldwide for 36 Cancers in 185 Countries. CA. Cancer J. Clin..

[3] Picon H., Guddati A.K. (2020). Mechanisms of Resistance in Head and Neck Cancer. Am. J. Cancer Res..

[4] Pontes F., Garcia A.R., Domingues I., João Sousa M., Felix R., Amorim C., Salgueiro F., Mariano M., Teixeira M. (2021). Survival Predictors and Outcomes of Patients with Recurrent and/or Metastatic Head and Neck Cancer Treated with Chemotherapy plus Cetuximab as First-Line Therapy: A Real-World Retrospective Study. Cancer Treat. Res. Commun..

[5] Vermorken J.B., Mesia R., Rivera F., Remenar E., Kawecki A., Rottey S., Erfan J., Zabolotnyy D., Kienzer H.-R., Cupissol D. (2008). Platinum-Based Chemotherapy plus Cetuximab in Head and Neck Cancer. N. Engl. J. Med..

[6] Rivera F., García-Castaño A., Vega N., Vega-Villegas M.E., Gutiérrez-Sanz L. (2009). Cetuximab in Metastatic or Recurrent Head and Neck Cancer: The EXTREME Trial. Expert Rev. Anticancer Ther..

[7] Muraro E., Fanetti G., Lupato V., Giacomarra V., Steffan A., Gobitti C., Vaccher E., Franchin G. (2021). Cetuximab in Locally Advanced Head and Neck Squamous Cell Carcinoma: Biological Mechanisms Involved in Efficacy, Toxicity and Resistance. Crit. Rev. Oncol. Hematol..

[8] Coliat P., Ramolu L., Jégu J., Gaiddon C., Jung A.C., Pencreach E. (2019). Constitutive or Induced HIF-2 Addiction Is Involved in Resistance to Anti-EGFR Treatment and Radiation Therapy in HNSCC. Cancers.

[9] Job S., de Reyniès A., Heller B., Weiss A., Guérin E., Macabre C., Ledrappier S., Bour C., Wasylyk C., Etienne-Selloum N. (2019). Preferential Response of Basal-Like Head and Neck Squamous Cell Carcinoma Cell Lines to EGFR-Targeted Therapy Depending on EREG-Driven Oncogenic Addiction. Cancers.

[10] Licona C., Delhorme J.-B., Riegel G., Vidimar V., Cerón-Camacho R., Boff B., Venkatasamy A., Tomasetto C., da Silva Figueiredo Celestino Gomes P., Rognan D. (2020). Anticancer Activity of Ruthenium and Osmium Cyclometalated Compounds: Identification of ABCB1 and EGFR as Resistance Mechanisms. Inorg. Chem. Front..

[11] Alsahafi E., Begg K., Amelio I., Raulf N., Lucarelli P., Sauter T., Tavassoli M. (2019). Clinical Update on Head and Neck Cancer: Molecular Biology and Ongoing Challenges. Cell Death Dis..

[12] Ortiz-Cuaran S., Bouaoud J., Karabajakian A., Fayette J., Saintigny P. (2021). Precision Medicine Approaches to Overcome Resistance to Therapy in Head and Neck Cancers. Front. Oncol..

[13] Ferris R.L., Blumenschein G., Fayette J., Guigay J., Colevas A.D., Licitra L., Harrington K., Kasper S., Vokes E.E., Even C. (2016). Nivolumab for Recurrent Squamous-Cell Carcinoma of the Head and Neck. N. Engl. J. Med..

[14] Seiwert T.Y., Burtness B., Mehra R., Weiss J., Berger R., Eder J.P., Heath K., McClanahan T., Lunceford J., Gause C. (2016). Safety and Clinical Activity of Pembrolizumab for Treatment of Recurrent or Metastatic Squamous Cell Carcinoma of the Head and Neck (KEYNOTE-012): An Open-Label, Multicentre, Phase 1b Trial. Lancet Oncol..

[15] Bagaev A., Kotlov N., Nomie K., Svekolkin V., Gafurov A., Isaeva O., Osokin N., Kozlov I., Frenkel F., Gancharova O. (2021). Conserved Pan-Cancer Microenvironment Subtypes Predict Response to Immunotherapy. Cancer Cell.

[16] Mueller C.G., Gaiddon C., Venkatasamy A. (2021). Current Clinical and Pre-Clinical Imaging Approaches to Study the Cancer-Associated Immune System. Front. Immunol..

[17] Dunn G.P., Old L.J., Schreiber R.D. (2004). The Three Es of Cancer Immunoediting. Annu. Rev. Immunol..

[18] Munhoz R.R., Postow M.A. (2016). Recent Advances in Understanding Antitumor Immunity. F1000Research.

[19] Markov O.V., Mironova N.L., Vlasov V.V., Zenkova M.A. (2016). Molecular and Cellular Mechanisms of Antitumor Immune Response Activation by Dendritic Cells. Acta Naturae.

[20] Elmusrati A., Wang J., Wang C.-Y. (2021). Tumor Microenvironment and Immune Evasion in Head and Neck Squamous Cell Carcinoma. Int. J. Oral Sci..

[21] Wan Y.Y. (2010). Regulatory T Cells: Immune Suppression and Beyond. Cell. Mol. Immunol..

[22] Pan Y., Yu Y., Wang X., Zhang T. (2020). Tumor-Associated Macrophages in Tumor Immunity. Front. Immunol..

[23] Yang Y., Li C., Liu T., Dai X., Bazhin A.V. (2020). Myeloid-Derived Suppressor Cells in Tumors: From Mechanisms to Antigen Specificity and Microenvironmental Regulation. Front. Immunol..

[24] Solomon B., Young R.J., Rischin D. (2018). Head and Neck Squamous Cell Carcinoma: Genomics and Emerging Biomarkers for Immunomodulatory Cancer Treatments. Semin. Cancer Biol..

[25] Galluzzi L., Vitale I., Warren S., Adjemian S., Agostinis P., Martinez A.B., Chan T.A., Coukos G., Demaria S., Deutsch E. (2020). Consensus Guidelines for the Definition, Detection and Interpretation of Immunogenic Cell Death. J. Immunother. Cancer.

[26] Kepp O., Senovilla L., Vitale I., Vacchelli E., Adjemian S., Agostinis P., Apetoh L., Aranda F., Barnaba V., Bloy N. (2014). Consensus Guidelines for the Detection of Immunogenic Cell Death. Oncoimmunology.

[27] Galluzzi L., Vitale I., Aaronson S.A., Abrams J.M., Adam D., Agostinis P., Alnemri E.S., Altucci L., Amelio I., Andrews D.W. (2018). Molecular Mechanisms of Cell Death: Recommendations of the Nomenclature Committee on Cell Death 2018. Cell Death Differ..

[28] Limagne E., Nuttin L., Thibaudin M., Jacquin E., Aucagne R., Bon M., Revy S., Barnestein R., Ballot E., Truntzer C. (2022). MEK Inhibition Overcomes Chemoimmunotherapy Resistance by Inducing CXCL10 in Cancer Cells. Cancer Cell.

[29] Jung A.C., Moinard-Butot F., Thibaudeau C., Gasser G., Gaiddon C. (2021). Antitumor Immune Response Triggered by Metal-Based Photosensitizers for Photodynamic Therapy: Where Are We?. Pharmaceutics.

[30] Pozzi C., Cuomo A., Spadoni I., Magni E., Silvola A., Conte A., Sigismund S., Ravenda P.S., Bonaldi T., Zampino M.G. (2016). The EGFR-Specific Antibody Cetuximab Combined with Chemotherapy Triggers Immunogenic Cell Death. Nat. Med..

[31] Park S.-J., Ye W., Xiao R., Silvin C., Padget M., Hodge J.W., van Waes C., Schmitt N.C. (2019). Cisplatin and Oxaliplatin Induce Similar Immunogenic Changes in Preclinical Models of Head and Neck Cancer. Oral Oncol..

[32] Tissue-Specific Transcription Factor Pit-1/GHF-1 Binds to the c-Fos Serum Response Element and Activates c-Fos Transcription. Molecular Endocrinology. Oxford Academic. https://academic.oup.com/mend/article/13/5/742/2741697.

[33] Gottardi C.J., Dunbar L.A., Caplan M.J. (1995). Biotinylation and Assessment of Membrane Polarity: Caveats and Methodological Concerns. Am. J. Physiol.-Ren. Physiol..

[34] Obeid M., Tesniere A., Ghiringhelli F., Fimia G.M., Apetoh L., Perfettini J.-L., Castedo M., Mignot G., Panaretakis T., Casares N. (2007). Calreticulin Exposure Dictates the Immunogenicity of Cancer Cell Death. Nat. Med..

[35] Blanchet A., Bourgmayer A., Kurtz J.-E., Mellitzer G., Gaiddon C. (2021). Isoforms of the P53 Family and Gastric Cancer: A Ménage à Trois for an Unfinished Affair. Cancers.

[36] Di Agostino S., Sorrentino G., Ingallina E., Valenti F., Ferraiuolo M., Bicciato S., Piazza S., Strano S., del Sal G., Blandino G. (2016). YAP Enhances the Pro-Proliferative Transcriptional Activity of Mutant P53 Proteins. EMBO Rep..

[37] Donehower L.A., Soussi T., Korkut A., Liu Y., Schultz A., Cardenas M., Li X., Babur O., Hsu T.-K., Lichtarge O. (2019). Integrated Analysis of TP53 Gene and Pathway Alterations in The Cancer Genome Atlas. Cell Rep..

[38] Gaiddon C., Lokshin M., Ahn J., Zhang T., Prives C. (2001). A Subset of Tumor-Derived Mutant Forms of P53 Down-Regulate P63 and P73 through a Direct Interaction with the P53 Core Domain. Mol. Cell. Biol..

[39] Miller J.J., Gaiddon C., Storr T. (2020). A Balancing Act: Using Small Molecules for Therapeutic Intervention of the P53 Pathway in Cancer. Chem. Soc. Rev..

[40] Miller J.J., Blanchet A., Orvain C., Nouchikian L., Reviriot Y., Clarke R.M., Martelino D., Wilson D., Gaiddon C., Storr T. (2019). Bifunctional Ligand Design for Modulating Mutant P53 Aggregation in Cancer. Chem. Sci..

[41] Apetoh L., Ghiringhelli F., Tesniere A., Obeid M., Ortiz C., Criollo A., Mignot G., Maiuri M.C., Ullrich E., Saulnier P. (2007). Toll-like Receptor 4–Dependent Contribution of the Immune System to Anticancer Chemotherapy and Radiotherapy. Nat. Med..

[42] Tokunaga R., Zhang W., Naseem M., Puccini A., Berger M.D., Soni S., McSkane M., Baba H., Lenz H.-J. (2018). CXCL9, CXCL10, CXCL11/CXCR3 Axis for Immune Activation—A Target for Novel Cancer Therapy. Cancer Treat. Rev..

[43] Sung F.L., Poon T.C.W., Hui E.P., Ma B.B.Y., Liong E., To K.F. (2005). Antitumor Effect and Enhancement of Cytotoxic Drug Activity by Cetuximab in Nasopharyngeal Carcinoma Cells. In Vivo.

[44] Son D.J., Hong J.E., Ban J.O., Park J.H., Lee H.L., Gu S.M., Hwang J.Y., Jung M.H., Lee D.W., Han S.-B. (2015). Synergistic Inhibitory Effects of Cetuximab and Cisplatin on Human Colon Cancer Cell Growth via Inhibition of the ERK-Dependent EGF Receptor Signaling Pathway. BioMed Res. Int..

[45] Jiang N., Wang D., Hu Z., Shin H., Qian G., Rahman M., Zhang H., Amin A., Nannapaneni S., Wang X. (2014). Combination of Anti-HER3 Antibody MM-121/SAR256212 and Cetuximab Inhibits Tumor Growth in Preclinical Models of Head and Neck Squamous Cell Carcinoma. Mol. Cancer Ther..

[46] Hientz K., Mohr A., Bhakta-Guha D., Efferth T. (2017). The Role of P53 in Cancer Drug Resistance and Targeted Chemotherapy. Oncotarget.

[47] Zhou G., Liu Z., Myers J.N. (2016). TP53 Mutations in Head and Neck Squamous Cell Carcinoma and Their Impact on Disease Progression and Treatment Response. J. Cell. Biochem..

[48] Jiang Z., Liu Z., Li M., Chen C., Wang X. (2018). Immunogenomics Analysis Reveals That TP53 Mutations Inhibit Tumor Immunity in Gastric Cancer. Transl. Oncol..

[49] Zhang H., Huang Z., Song Y., Yang Z., Shi Q., Wang K., Zhang Z., Liu Z., Cui X., Li F. (2021). The TP53-Related Signature Predicts Immune Cell Infiltration, Therapeutic Response, and Prognosis in Patients with Esophageal Carcinoma. Front. Genet..

[50] Guo G., Yu M., Xiao W., Celis E., Cui Y. (2017). Local Activation of P53 in the Tumor Microenvironment Overcomes Immune Suppression and Enhances Antitumor Immunity. Cancer Res..

[51] Deb S.P., Muñoz R.M., Brown D.R., Subler M.A., Deb S. (1994). Wild-Type Human P53 Activates the Human Epidermal Growth Factor Receptor Promoter. Oncogene.

[52] Ludes-Meyers J.H., Subler M.A., Shivakumar C.V., Munoz R.M., Jiang P., Bigger J.E., Brown D.R., Deb S.P., Deb S. (1996). Transcriptional Activation of the Human Epidermal Growth Factor Receptor Promoter by Human P53. Mol. Cell. Biol..

[53] Sauer L., Gitenay D., Vo C., Baron V.T. (2010). Mutant P53 Initiates a Feedback Loop That Involves Egr-1/EGF Receptor/ERK in Prostate Cancer Cells. Oncogene.

[54] Huang S., Benavente S., Armstrong E.A., Li C., Wheeler D.L., Harari P.M. (2011). P53 Modulates Acquired Resistance to EGFR Inhibitors and Radiation. Cancer Res..

[55] Bouali S., Chrétien A.-S., Ramacci C., Rouyer M., Marchal S., Galenne T., Juin P., Becuwe P., Merlin J.-L. (2009). P53 and PTEN Expression Contribute to the Inhibition of EGFR Downstream Signaling Pathway by Cetuximab. Cancer Gene Ther..

[56] Faridoni-Laurens L., Tourpin S., Alsafadi S., Barrois M., Temam S., Janot F., Koscielny S., Bosq J., Bénard J., Ahomadegbe J.-C. (2008). Involvement of N-Terminally Truncated Variants of P73, DeltaTAp73, in Head and Neck Squamous Cell Cancer: A Comparison with P53 Mutations. Cell Cycle Georget. Tex.

[57] Gwosdz C., Balz V., Scheckenbach K., Bier H., Bier H. (2004). P53, P63 and P73 Expression in Squamous Cell Carcinomas of the Head and Neck and Their Response to Cisplatin Exposure. Advances in Oto-Rhino-Laryngology.

[58] Knickelbein K., Tong J.-S., Chen D., Wang Y.-J., Misale S., Bardelli A., Yu J., Zhang L. (2018). Restoring PUMA Induction Overcomes KRAS-Mediated Resistance to Anti-EGFR Antibodies in Colorectal Cancer. Oncogene.

[59] Rocco J.W., Leong C.-O., Kuperwasser N., DeYoung M.P., Ellisen L.W. (2006). P63 Mediates Survival in Squamous Cell Carcinoma by Suppression of P73-Dependent Apoptosis. Cancer Cell.

[60] Rocco J.W. (2006). p63 and p73: Life and Death in Squamous Cell Carcinoma. Cell Cycle.

[61] Panaretakis T., Kepp O., Brockmeier U., Tesniere A., Bjorklund A.-C., Chapman D.C., Durchschlag M., Joza N., Pierron G., van Endert P. (2009). Mechanisms of Pre-Apoptotic Calreticulin Exposure in Immunogenic Cell Death. EMBO J..

[62] Martins I., Kepp O., Schlemmer F., Adjemian S., Tailler M., Shen S., Michaud M., Menger L., Gdoura A., Tajeddine N. (2011). Restoration of the Immunogenicity of Cisplatin-Induced Cancer Cell Death by Endoplasmic Reticulum Stress. Oncogene.

[63] Sims G.P., Rowe D.C., Rietdijk S.T., Herbst R., Coyle A.J. (2010). HMGB1 and RAGE in Inflammation and Cancer. Annu. Rev. Immunol..

[64] Sistigu A., Yamazaki T., Vacchelli E., Chaba K., Enot D.P., Adam J., Vitale I., Goubar A., Baracco E.E., Remédios C. (2014). Cancer Cell–Autonomous Contribution of Type I Interferon Signaling to the Efficacy of Chemotherapy. Nat. Med..

[65] Judd N.P., Allen C.T., Winkler A.E., Uppaluri R. (2012). Comparative Analysis of Tumor Infiltrating Lymphocytes in a Syngeneic Mouse Model of Oral Cancer. Octolaryngol.-Head Neck Surg..

[66] Garrido G., Rabasa A., Sánchez B., López M.V., Blanco R., López A., Hernández D.R., Pérez R., Fernández L.E. (2011). Induction of Immunogenic Apoptosis by Blockade of Epidermal Growth Factor Receptor Activation with a Specific Antibody. J. Immunol. Baltim. Md 1950.

[67] Galluzzi L., López-Soto A., Kumar S., Kroemer G. (2016). Caspases Connect Cell-Death Signaling to Organismal Homeostasis. Immunity.

[68] Wu J., Waxman D.J. (2018). Immunogenic Chemotherapy: Dose and Schedule Dependence and Combination with Immunotherapy. Cancer Lett..

[69] Tatarova Z., Blumberg D.C., Korkola J.E., Heiser L.M., Muschler J.L., Schedin P.J., Ahn S.W., Mills G.B., Coussens L.M., Jonas O. (2022). A Multiplex Implantable Microdevice Assay Identifies Synergistic Combinations of Cancer Immunotherapies and Conventional Drugs. Nat. Biotechnol..

