# Supplementary material for: The EXTREME Regimen Associating Cetuximab and Cisplatin Favors Head and Neck Cancer Cell Death and Immunogenicity with the Induction of an Anti-Cancer Immune Response
Source: Cells. 2022 Sep 14;11(18):2866. doi: 10.3390/cells11182866 (PMC9496761; doi:10.3390/cells11182866)
Supplement: Supplementary file 1 [file cells-11-02866-s001.zip › cells-1874860-Supplementary Figures.pdf]

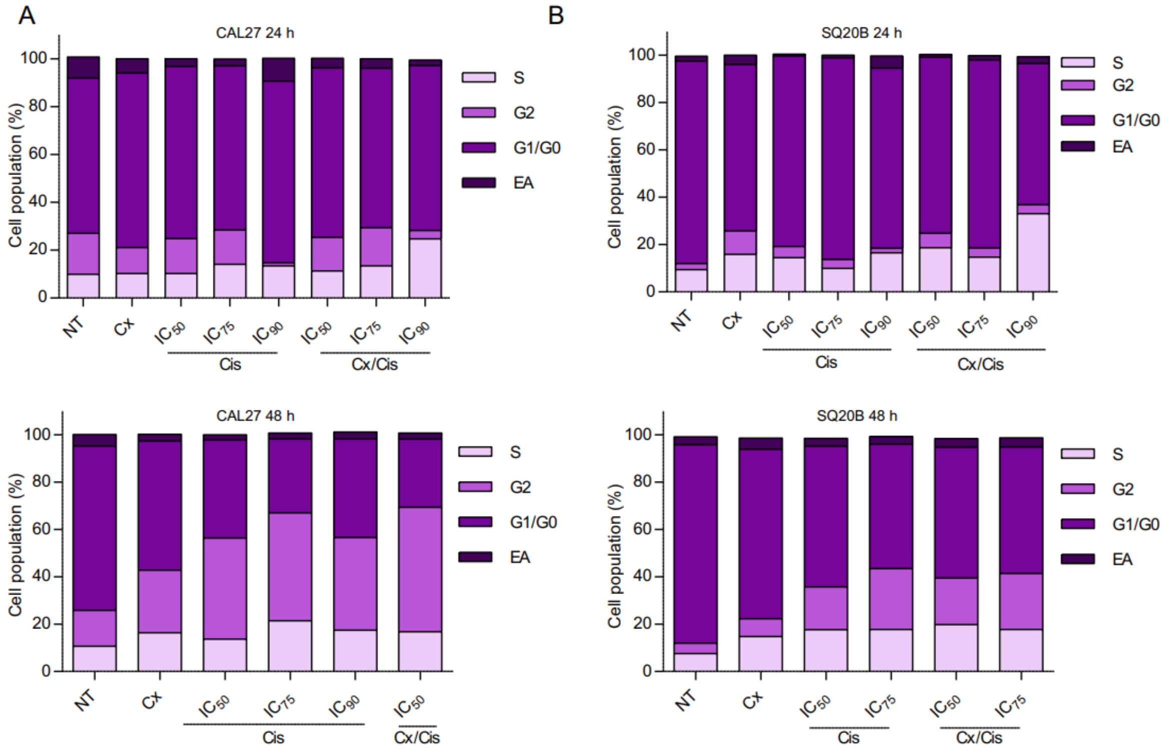

Figure S1. A,B. Quantification of the cell cycle distribution data of CAL27 (A) and SQ20B (B) cells treated 24 h (upper histograms) and 48 h (lower histograms) with cetuximab (Cx), cisplatin (Cis) at the IC50 and IC75 and the Cx/Cis combination. The histograms show the mean number of percentages of cells in S, G2, G1/G0, and early apoptosis (EA; i.e., sub-G1).;.

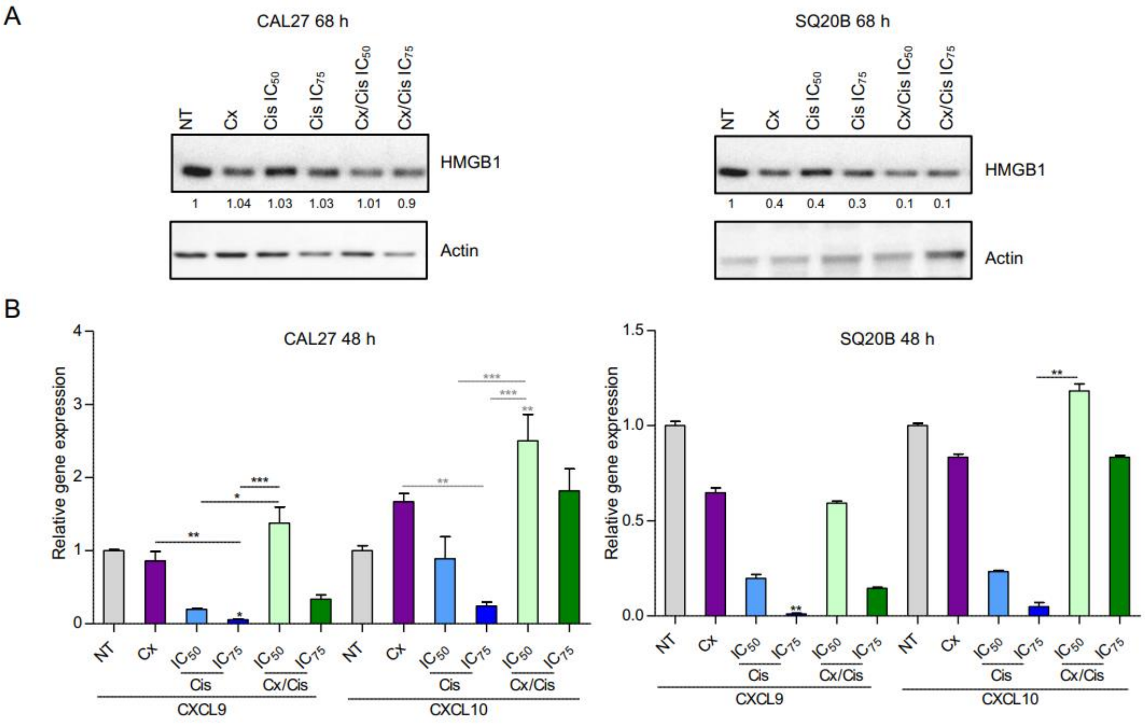

Figure S2. A. Western blot analysis of the HMGB1 expression in the supernatant from CAL27 (left panels) and SQ20B (right panels) cell cultures

harvested 68 h after with cetuximab (Cx), cisplatin (Cis) at the IC50 and IC75 and the Cx/Cis combination. Signals were quantified respectively to the actin loading Cells 2022, 11, x FOR PEER REVIEW 21 of 24 control and normalized with respect to the non-treated control (quantification results are shown). The blots that are shown are re Apresentative examples of three independent experiments. Additional independent biological replicates are shown in supporting documents. B. Analysis of the expression of CXCL9 and CXCL10 by RT-qPCR in CAL27 (left histographs) and SQ20B (right histographs) cells treated for 48 h with cetuximab (Cx), cisplatin (Cis) at the IC50 and IC75 and the Cx/Cis combination. Data are represented as the mean from one independent experiment +SEM;.

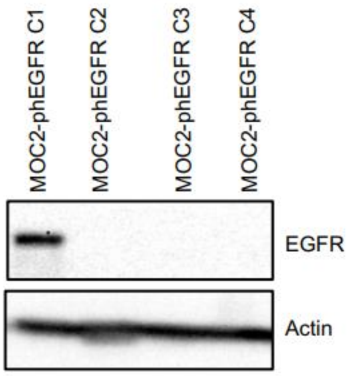

Figure S3. Western blot analysis of EGFR expression in whole-cell protein extracts from MOC-2 cells transduced with a pBABE-hEGFR expression plasmid after selection of clones on puromycin. EGFR expression is observed in the MOC2-phEGFR C1 clone. The blots that are shown are representative examples of three independent experiments;.

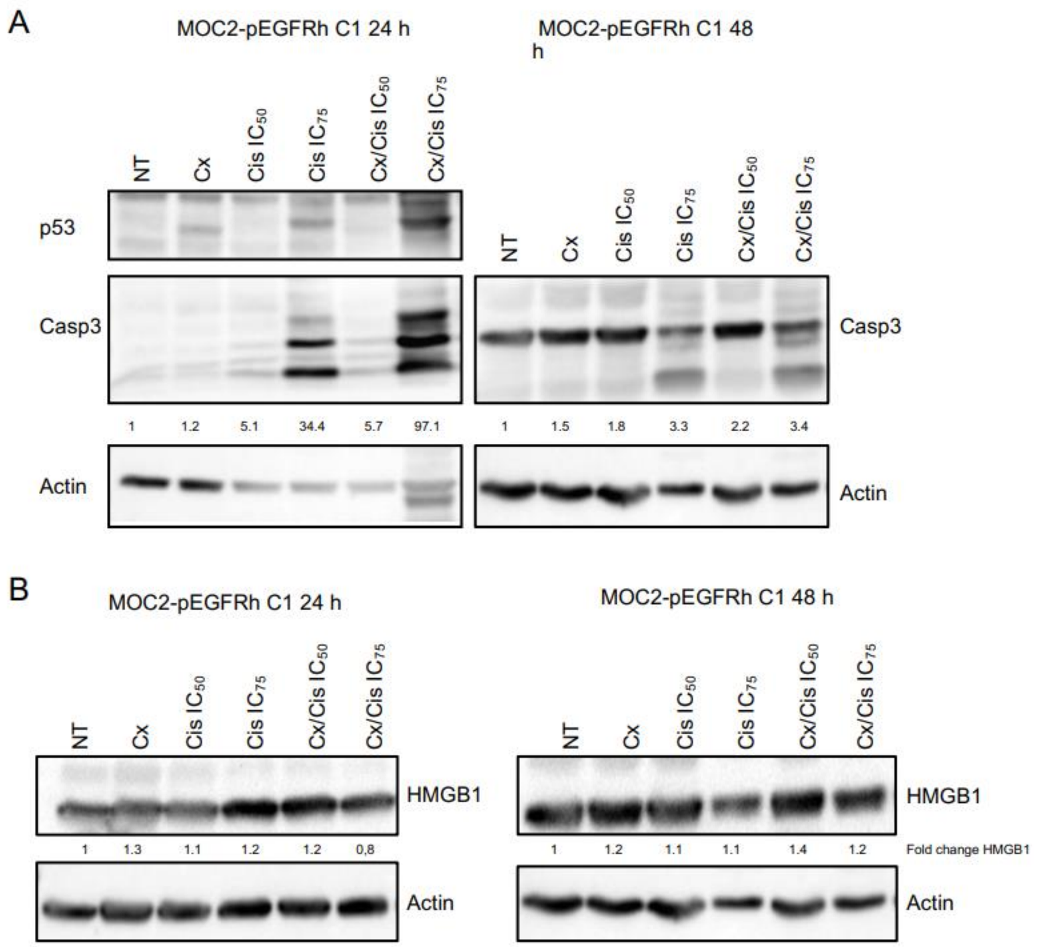

Figure S4. A. Western blot analysis of p53 and cleaved caspase-3 (Casp3) expression in whole protein extracts from MOC2-phEGFR C1 cells treated with cetuximab (Cx), cisplatin (Cis) at the IC50, IC75, and IC90, and the Cx/Cis combination for 24 h (left panels) and 48 h (right panels). Casp3 signals were quantified respectively to the actin loading control and normalized with respect to the non-treated control (quantification results are shown). B. Western blot analysis of the HMGB1 expression in whole protein extracts from MOC2-phEGFR C1 cell cultures treated for 24 h (left panels) and 48 h (right panels) with cetuximab (Cx), cisplatin (Cis) at the IC50, and IC75 and the Cx/Cis combination. Signals were quantified respectively to the actin loading control and normalized with respect to the non-treated control (quantification results are shown). Blots. shown are representative examples of two independent experiments. Additional independent biological replicates are shown in supporting documents.;.
