# Supplementary material for: The EXTREME Regimen Associating Cetuximab and Cisplatin Favors Head and Neck Cancer Cell Death and Immunogenicity with the Induction of an Anti-Cancer Immune Response
Source: Cells. 2022 Sep 14;11(18):2866. doi: 10.3390/cells11182866 (PMC9496761; doi:10.3390/cells11182866)
Supplement: Supplementary file 1 [file cells-11-02866-s001.zip › cells-1874860-Supplementary Tables.pdf]

Supplementary tables

Table S1. List of oligonucleotides primers used for RT-qPCR gene expression assays

Gene name	Forward primer (5'-3')	Reverse primer (5'-3')
DDB2	TGGCATCAGTTCGCTTAATG	ACTTCCGTGTCCTGGCTTC
FDRX2	TCCTACTGACCCCACCTGAG	TCGACTCTGCCTCAGTACACC
RPS27L	CAGATCGCTTGCAGCTTG	TCTTCCAAGGACGGATGTAGTAA
ZMAT3	GCCAGGAAAGAAGGGAATG	GCGGGGATTGAAGTAAGGAC
RPLPO	GAAGGCTGTGGTGCTGATGG	CCGGATATGAGGCAGCAGTT
GAPDH	GCACAAGAGGAAGAGAGAGACC	AGGGGAGATTCAGTGTGGTG

Gene names, forward and reverse primer sequences are shown

Table S2. List of TaqMan assays used for RT-qPCR gene expression assays

Gene name	TaqMan gene expression assay ID
CXCL9	Hs00171065_m1
CXC10	Hs00171042_m1
TBP	Hs00427620_m1

Gene names and TaqMan gene expression assay ID are shown

Table S3. List of antibodies used for western blot analysis

Protein	Provider	Antibody dilution
Cleaved caspase-3	Asp175 Cell signaling	1/1000
p53	DO-1 Santa cruz	1/1000
p63	4A4 Abcam	1/1000
p73	EP436Y Abcam	1/1000
Calreticulin	D3E6 Cell signaling	1/1000
EGFR	D38B1 Cell signaling	1/2000
HMGB1	1856 abcam	1/1000 (supernatant)<br>1/5000 (whole cell extract)
Actin	C4	1/10 000
GAPDH	House made	1/000
BSA	B2901 sigma	1/1000

Protein names, antibody providers and used antibody dilutions are shown.
